# Characterization of Anti-Inflammatory and Anti-Proliferative Triterpenoids and Phytosterols from Cranberry Pomace

**DOI:** 10.3390/molecules31162853

**Published:** 2026-08-15

**Authors:** Md Sagir Mia, Huifang Li, Ying Chen, Tracie Ferreira, Md Afjalus Siraj, Muaz Faruque, Hang Ma, Christina Khoo, Lindsey Christman, Catherine Neto

**Affiliations:** 1 Department of Chemistry and Biochemistry, University of Massachusetts Dartmouth, North Dartmouth, MA 02747, USA; mmia@umassd.edu; 2 Bioactive Botanical Research Laboratory, Department of Biomedical and Pharmaceutical Sciences, College of Pharmacy, University of Rhode Island, Kingston, RI 02881, USA; huifang_li@uri.edu (H.L.); hang_ma@uri.edu (H.M.); 3 Department of Obstetrics and Gynecology, The Second Affiliated Hospital of Soochow University, Suzhou 215004, China; 4 Department of Bioengineering, University of Massachusetts Dartmouth, North Dartmouth, MA 02747, USA; tferreira@umassd.edu; 5 Department of Pharmaceutical Sciences, College of Pharmacy, Ferris State University, Big Rapids, MI 49307, USA; mdafjalussiraj@ferris.edu; 6 Department of Therapeutic Radiology, Yale School of Medicine, Yale University, New Haven, CT 06511, USA; muaz.faruque@yale.edu; 7 Ocean Spray Cranberries, Inc., Lakeville, MA 02349, USA; ckhoo@oceanspray.com (C.K.); lchristman@oceanspray.com (L.C.)

**Keywords:** cranberry pomace, triterpenoids, phytosterols, UPLC-MS, GC-MS, anti-inflammatory, anti-proliferative, lipoxygenase, THP-1, inflammasome, molecular docking

## Abstract

*Vaccinium macrocarpon* (American Cranberry) fruit is processed to make juice, supplements, and other edible products, leaving pomace as a sidestream that contains secondary metabolites with potential anti-inflammatory and anti-proliferative activities. Ultrasound-assisted extraction and chromatographic methods were developed to prepare an extract (POM-ACE) and fractions rich in pentacyclic triterpenoids and phytosterols for bioactivity evaluation. These were characterized using UPLC-MS and GC-MS. Anti-inflammatory effects were assessed in a human monocyte (THP-1) model, identifying several fractions containing triterpenoids and sitosterol that significantly inhibited IL-1β expression. Molecular docking indicated favorable interactions of these components with NLRP3, suggesting a possible modulation of inflammasome. Fractions rich in ursolic acid (UA) and oleanolic acid (OA) and their p-hydroxycinnamic acid (HCA) esters also showed mild concentration-dependent inhibition of lipoxygenase (LOX) activity. Pearson correlation analysis identified strong positive correlations between UA, OA, and their trans-HCA esters with LOX inhibition. Anti-proliferative activity was assessed in HT-29 colon adenocarcinoma cells using MTT, with several fractions exhibiting moderate concentration-dependent activity (IC_50_ = 12–17 µg/mL). PLS regression analysis supported significant contributions by UA, OA, and their HCA esters. Flow cytometry experiments demonstrated that cell death occurs in part through apoptosis; the role of apoptosis was further supported by favorable molecular docking interactions between pomace triterpenoids and caspases-3 and -9. These findings suggest that cranberry pomace is a promising source of triterpenoids and phytosterols with anti-inflammatory and anti-proliferative properties.

## 1. Introduction

American cranberry (*Vaccinium macrocarpon*) is widely processed for juice, generating large quantities of pomace (skins, seeds, and peels) as a sidestream [1]. Although typically discarded, cranberry pomace, like other fruit pomaces, contains a large biomass of peels that are rich in phytochemicals, and may serve as sustainable sources of functional food ingredients [2,3,4,5,6,7]. An example would be triterpenoids, a component of cuticular wax. Triterpenoids are C_30_ metabolites derived from isopentenyl pyrophosphate oligomers, with more than 20,000 compounds identified in nature [2]. They exhibit diverse pharmacological activities, including anti-inflammatory, antioxidant, hepatoprotective, antimicrobial, and anti-cancer effects [8,9,10]. Triterpenoids that reduce the growth of multiple cancer cell lines have been isolated from the cuticular wax of lingonberry (*Vaccinium vitis-ideae*) [11]. Triterpenoids previously identified in cranberries, including ursolic acid (UA), oleanolic acid (OA), corosolic acid (CA), and maslinic acid (MA) have been reported to reduce oxidative stress, suppress NF-κB signaling, decrease the expression of pro-inflammatory mediators such as COX-2 and iNOS, and induce apoptosis in colon tumor cells without harming normal cells [12,13,14,15,16,17,18]. The most plentiful among these, UA, is reported to modulate the MAPK (mitogen-activated protein kinase) pathway in colon cancer cells [19] and osteosarcoma cells [20], and induce apoptosis through caspase activation in multiple cell lines [19,20,21].

Inflammation is the protective or immune response in the body against noxious stimuli like chemicals, ultraviolet, and microbes. Inflammation is a biological response of the immune system to a variety of factors, such as infection, injury, or toxic compounds, presented by pain, swelling, and increased temperature at the site of inflammation [22]. Excessive or chronic inflammation can initiate inflammation associated with diseases like cancer [23]. It involves arachidonic acid metabolism via the cyclooxygenase (COX), lipoxygenase (LOX), and cytochrome P450 (CYP) pathways, as well as inflammasome-mediated caspase-1 activation, which promotes IL-1β maturation [23,24,25]. Consequently, inhibition of LOX enzymes and inflammasome pathways has emerged as a promising strategy for cancer prevention and the control of inflammatory diseases.

**Figure 1:**
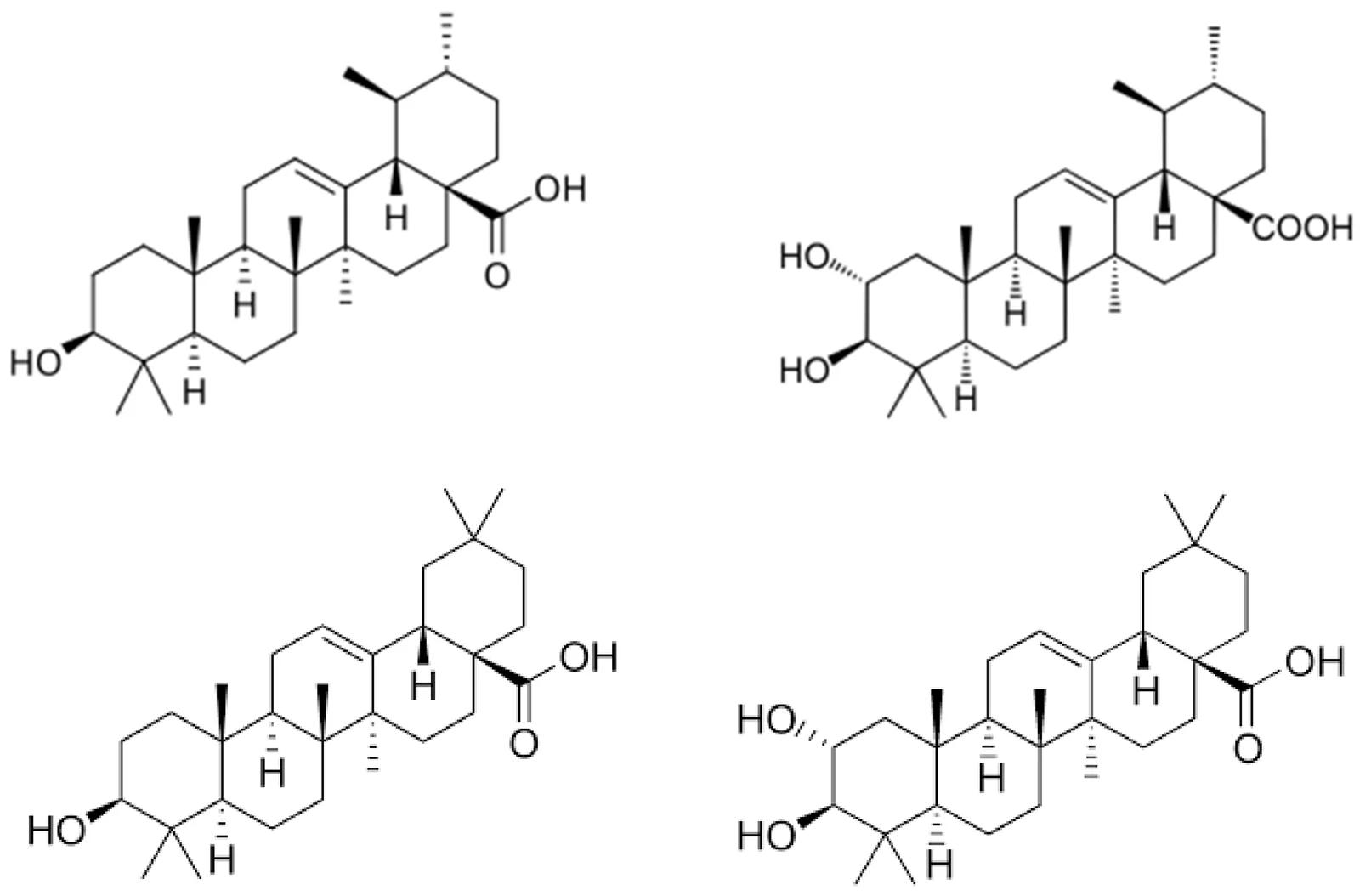
Major triterpenoid acids in pomace: ursolic acid (UA, **upper left**), corosolic acid (CA, **upper right**), oleanolic acid (OA, **lower left**), maslinic acid (MA, **lower right**).

**Figure 2:**
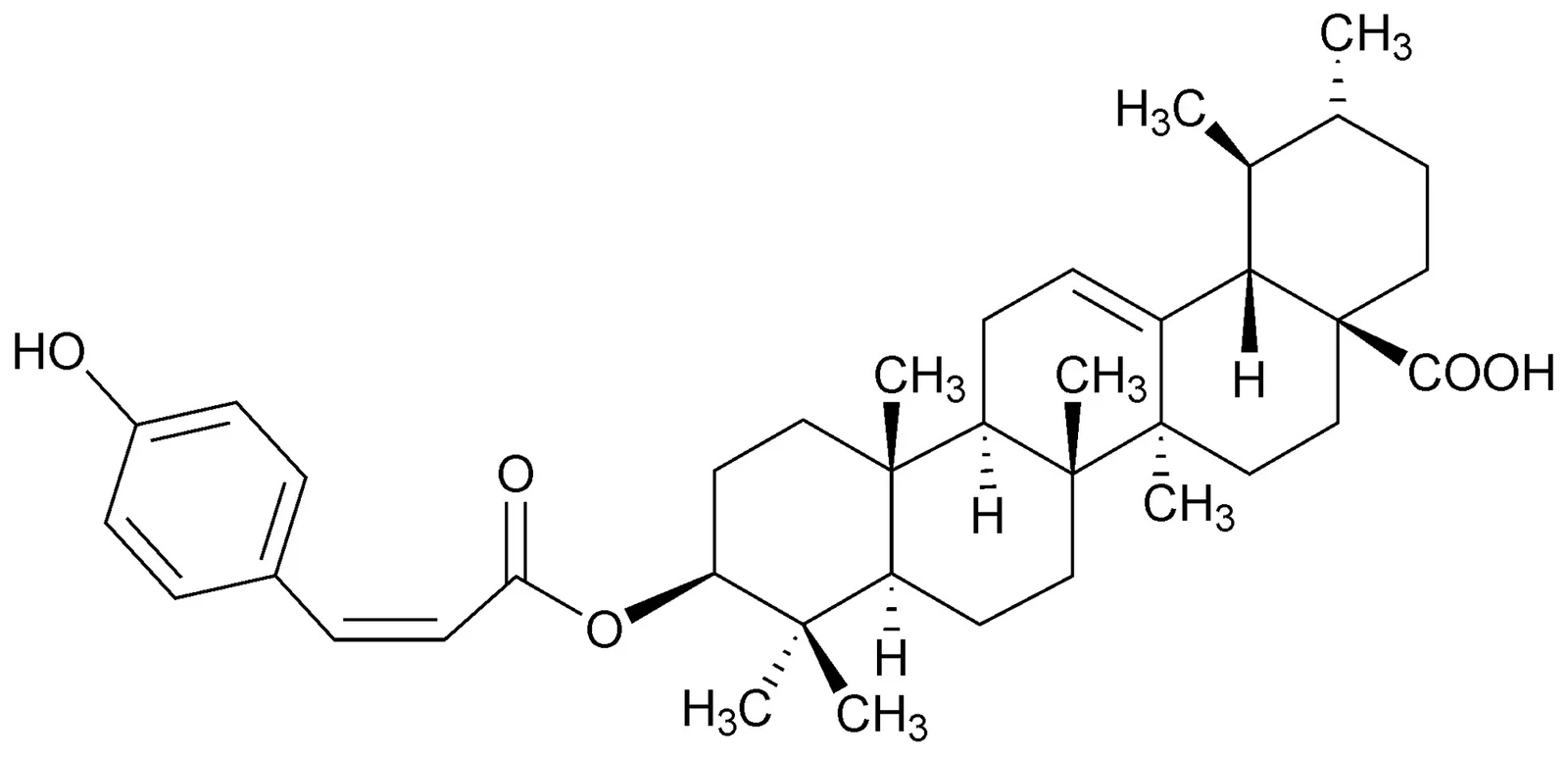
Triterpenoid esters previously identified in cranberry include *cis*-3-O-p-hydroxy-cinnamoylursolic acid (*cis*-HCA-UA, shown above) and its *trans*-isomer.

**Figure 3:**
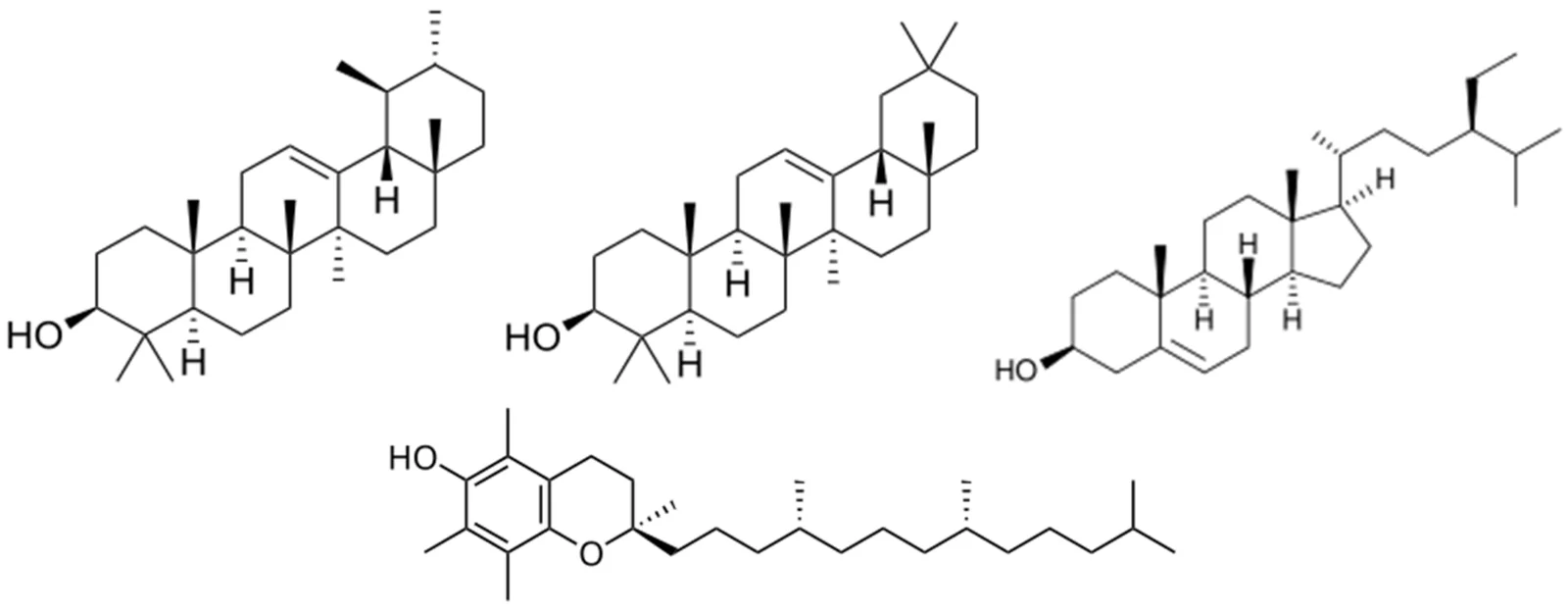
Neutral triterpenoids, sterols and tocopherols found in cranberry pomace include, top (left-to-right): α-amyrin, β-amyrin, β-sitosterol; bottom: α-tocopherol.

Cranberry fruits and raw materials have been reported to contain abundant UA and OA, as well as lesser amounts of MA and CA (shown in Figure 1) [3,4,5,6,7]. We have also previously reported abundant *cis*- and *trans*-hydroxycinnamoyl (HCA) esters of UA (Figure 2) and OA in cranberry pomace, press cake, and whole fruits [3]. Our earlier studies demonstrated that UA and its *cis*- and *trans*-3-O-p-hydroxycinnamoyl esters (Murphy) [12] can inhibit the growth of multiple cancer cell lines as well as inhibit the activity of matrix metalloproteinases (MMP-2 and MMP-9) in DU-145 cells, potentially limiting prostate carcinogenesis [13]. Other terpenoid constituents that may contribute to biological properties include α- and β-amyrin, sitosterol, other phytosterols, and tocopherols (Figure 3) [4,6]. Recently, we reported that a triterpenoid-rich extract of whole cranberry fruit containing UA and sitosterol significantly reduced colonic inflammation and tumorigenesis in a mouse model of colitis, and downregulated tissue expression of inflammatory cytokines IL-6, IL-1β, and TNF-α [26].

The goal of the present study was to evaluate cranberry pomace as a source of bioactive triterpenoids, with emphasis on their potential anti-inflammatory and anti-proliferative activities. We employed ultrasound-assisted extraction, and bioassay-guided fractionation to prepare triterpenoid-rich fractions from cranberry pomace, and developed reliable analysis methods to characterize bioactive triterpenoids, phytosterol and tocopherols present. These fractions were assessed for anti-proliferative activity in HT-29 colon adenocarcinoma cells. They were also evaluated for anti-inflammatory activities in a cellular model (THP-1 monocytes) and against lipoxygenase (LOX), an enzyme involved in the production of pro-inflammatory eicosanoids. Multivariate analyses were performed to correlate bioactivities to key pomace constituents and molecular docking experiments were used to assess their interactions with target proteins. Our findings suggest that several triterpenoids and beta-sitosterol in cranberry pomace play a role in these properties.

## 2. Results

### 2.1. Fractionation and Analysis

An ultrasound-assisted acetone extraction method was employed to maximize the yield of terpenes such as triterpenoids, sterols, carotenoids, and tocopherols, while minimizing the extraction of polyphenols. Chlorinated solvents such as dichloromethane or chloroform were avoided to comply with tightening regulations at our institution and EPA recommendations. Acetone afforded a broader triterpenoid acid profile than ethyl acetate. In total, 12.709 g POM-ACE solid extract powder was produced from 100 g of dried pomace. Based on LCMS and GCMS analysis, the POM-ACE contained ≥ 51% triterpenoids and ≥4% phytosterols. Triterpenoid acids in POM-ACE were primarily UA (263 mg/g dry weight), followed by OA (70.2 mg/g dry weight), CA (14.8 mg/g dry weight), and the *cis*- and *trans*-hydroxycinnamoyl esters of UA and OA (148 mg/g dry weight). All were identified and quantified using UPLC-MS. POM-ACE also contained neutral triterpenoids, including α-amyrin (5.69 mg/g dry weight) and β-amyrin (3.92 mg/g dry weight), as identified by GC-MS. POM-ACE also contained significant phytosterols, mainly β-sitosterol (28.1 mg/g dry weight) and campesterol (10.5 mg/g dry weight), identified and quantified using GC-MS. Fractionation of POM-ACE extract batch #1 (from 1 g extract) and batch #2 (from 5 g extract) produced semi-purified fractions A–M and 2A–2P, respectively. Tables S1 and S2 summarize solvent systems used for silica gel chromatography, purified fraction weights after pooling, and results of qualitative TLC analysis of the major fractions from the fractionations of 1 g and 5 g of pomace, respectively. Table 1 and Table 2 summarize bioactive analyte content based on UPLC-MS analysis of fractions A–M and 2A–2P, respectively. The content of sterols and neutral triterpenoids in pertinent fractions, as determined by GC-MS, is summarized in Table 3.

Reliable HPLC-DAD methods were also developed to identify and quantify triterpenoids and sterols in cranberry pomace extracts and their purified fractions (see Supplementary Materials Section S1). These methods can detect the major triterpenoid acids, their HCA esters (Figure S1), neutral triterpenoids and phytosterols in comparison to commercial standards, despite the lack of a strong chromophore. For greater sensitivity and accuracy, UPLC-MS with selected reaction monitoring was employed to determine triterpenoid acids and esters in POM-ACE and fractions using our previously developed UPLC-MS method [3]. The limits of quantification (LOQ) for UA, OA, MA, and CA were 0.0625 ppm, 0.0375 ppm, 0.01 ppm, and 0.025 ppm respectively; limits of detection (LOD) were 0.0187 ppm, 0.01125 ppm, 0.003 ppm, and 0.0075 ppm respectively.

UA is the major triterpenoid in pomace, followed by OA, CA, MA. β-sitosterol is the major phytosterol in pomace samples. Table 1 and Table 2 summarize the results of the quantitative analysis of triterpenoid acids and esters POM-ACE and its fractions using UPLC-MS. Table 3 summarizes the results of the quantitative analysis of major sterols, neutral triterpenoids and tocopherols present in POM-ACE and its fractions using GC-MS. Figure S2 shows the extracted ion chromatograms for LC-MS for the major triterpenoid acids and esters in POM-ACE, and Figure S3 shows the total ion chromatograms (TIC) for the major neutral triterpenoids, phytosterols, tocopherol in POM-ACE using GC-MS.

### 2.2. Biological Assay Data

Lipoxygenase inhibition data for selected fractions are summarized in Table 4. Fraction 2G exhibited moderate lipoxygenase inhibitory activity with IC_50_ = 104 µg/mL, followed by 2H at 136 µg/mL and K at 200 µg/mL. The other POM-ACE fractions tested were less effective LOX inhibitors in this assay. 2G and 2H showed stronger inhibition compared to POM-ACE. Fraction 2G contained nearly 100% triterpenoid acids and esters; the major constituents were UA (55%), OA (15%) and HCA esters of UA and OA (totaling 29%). The ester content of 2G was high compared to other fractions tested. Fraction 2H contained about 93% triterpenoids; UA (69%) and OA (12%) were the major constituents, followed by CA (5.6%) and MA (3%), which were present in greater amounts relative to other fractions tested.

**Figure 4:**
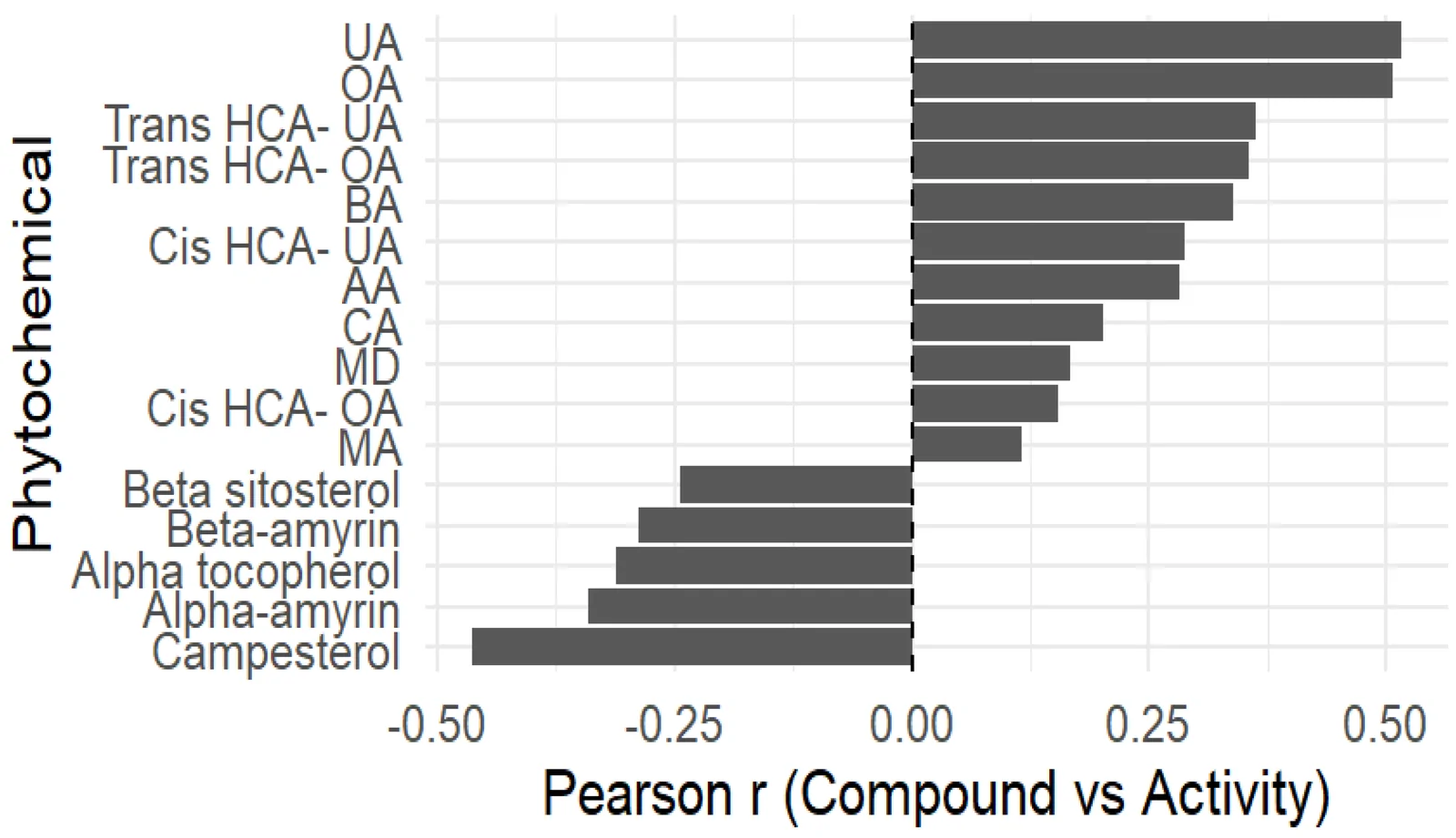
Correlation of phytochemical content in POM-ACE fractions with the fractions’ LOX inhibitory activity (IC_50_).

Pearson correlation analysis data correlating the phytochemical composition of selected fractions with the efficacy of LOX inhibition are shown in Figure 4. Positive correlations indicate that higher levels of certain compounds are associated with increased LOX inhibition, while negative ones suggest decreased activity. UA exhibits the strongest positive correlation with LOX inhibition, followed by OA and hydroxycinnamic acid derivatives *trans*-HCA–UA and *trans*-HCA–OA. These show moderate-to-strong positive Pearson correlation coefficients (r = 0.25–0.50), implying that samples rich in triterpenoid acids and HCA esters tend to have higher biological activity. Less plentiful components CA, MA, BA and trace components, asiatic acid (AA) and madecassic acid (MD), showed weaker but positive correlations, suggesting they may play secondary roles in activity without being primary drivers. In contrast, phytosterols and neutral triterpenoids, including β-sitosterol, α- and β-amyrin, α-tocopherol, and campesterol were negatively correlated with LOX inhibition. Overall, the data suggest that triterpenoid acids UA, OA and their HCA esters are the main chemical contributors to LOX inhibition activity, while sterols, amyrins, and tocopherols contribute minimally. Pearson correlation analysis confirms that UA, OA, and HCA esters are positively correlated with LOX inhibition. In contrast, phytosterols and amyrins are negatively associated.

**Figure 5:**
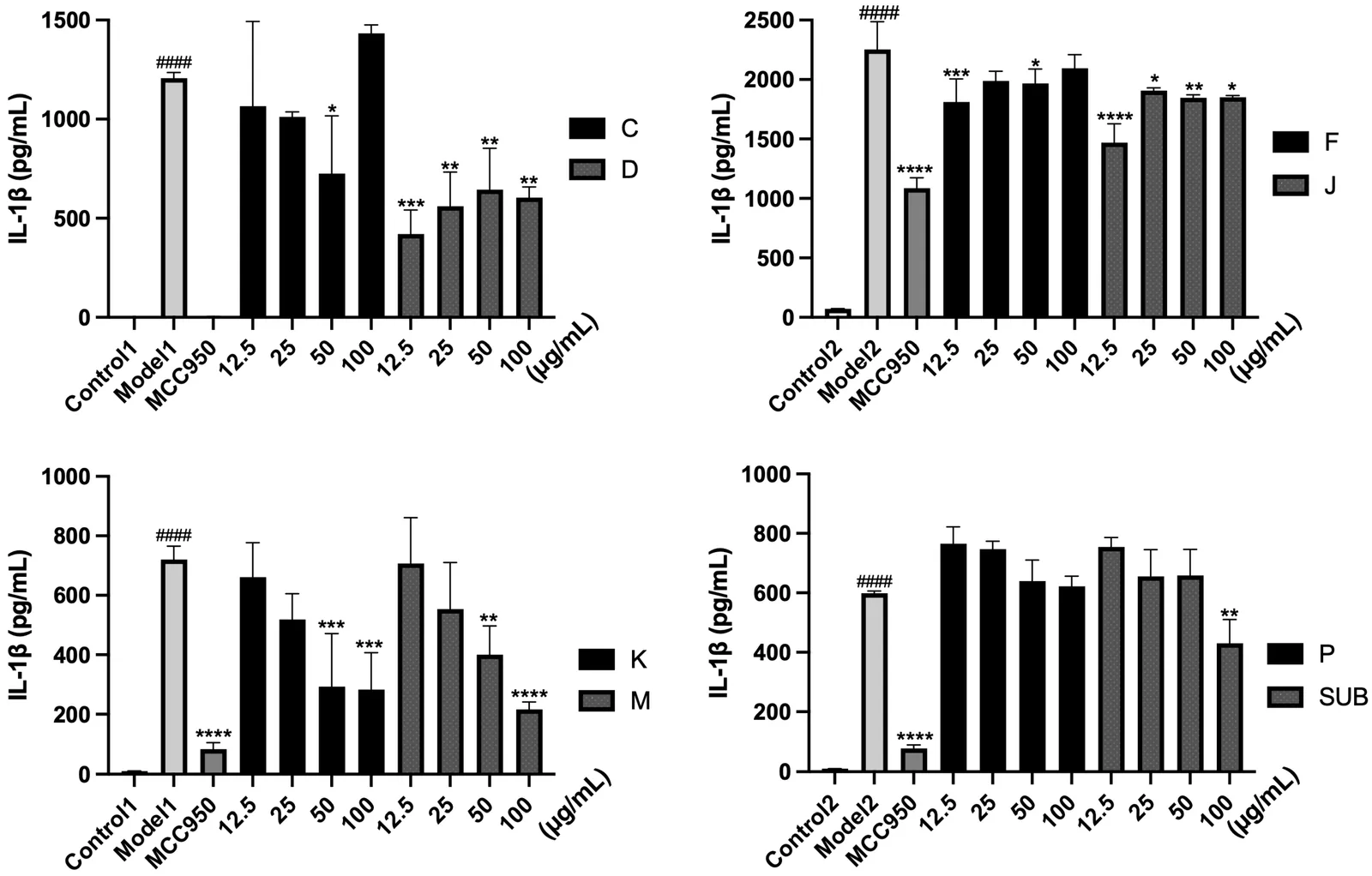
Inhibition of IL-1β secretion in LPS-stimulated THP-1 cells (model) by POM-ACE (denoted SUB, lower right) and fractions at 12.5–100 µg/mL, in comparison to positive controls (MCC950). Results represent the mean ± SD (*n* = 3); significant differences between treatments and model are denoted as * *p* < 0.05, ** *p* < 0.01, *** *p* < 0.001, **** *p* < 0.0001, as compared with the control group (marked ####).

Figure 5 shows the inhibition of IL-1β production in LPS-stimulated THP-1 cells when treated with POM-ACE (denoted SUB) and fractions at 12.5–100 µg/mL. POM-ACE extract reduced expression by about 30% at 100 µg/mL but was ineffective at lower concentrations. Among the fractions tested, fraction D was the most effective sample in reducing IL-1β levels at all test concentrations. A separate test found that treatment with D at concentrations as low as 1.25 µg/mL reduced IL-1β production (Figure S5). Fraction J showed inhibition at all test concentrations (12.5–100 µg/mL). Fractions K and M showed concentration-dependent inhibition of IL-1β production, and similar levels of activity, whereby inhibition was observed at 50–100 µg/mL. Fraction P was ineffective at all test concentrations.

**Figure 6:**
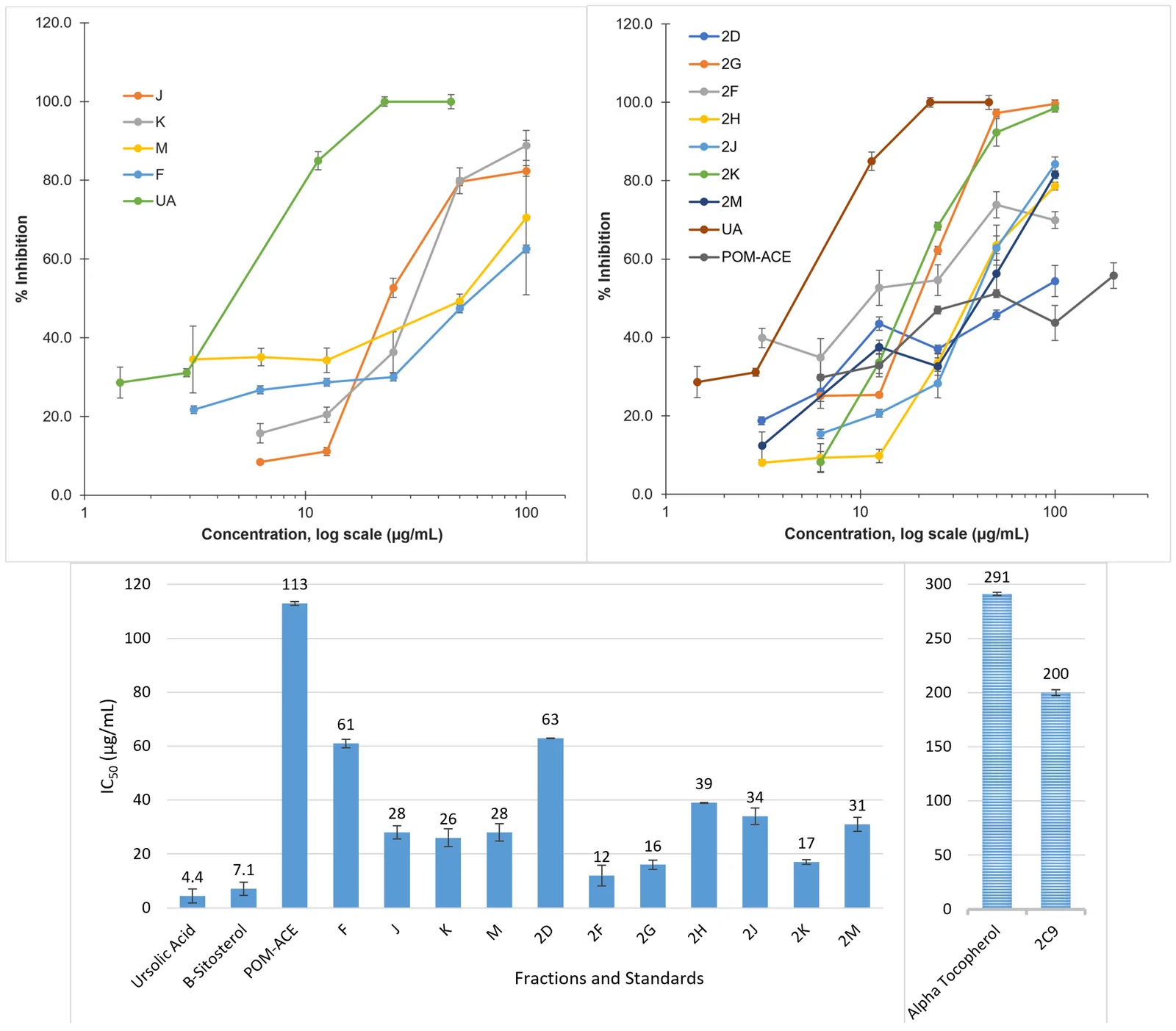
**Upper**: dose–response curves for 24 h treatment of HT-29 cells with POM-ACE, fractions and selected commercial standards (*n* = 3) based on MTT assay data. **Lower**: calculated mean IC_50_ ± SD (*n* = 3) for inhibition of HT-29 cell proliferation by POM-ACE, fractions and selected standards over 24 h, compared to untreated control.

Preliminary screenings for anti-proliferative activity of POM-ACE and its fractions were performed using MTT assay against HT-29 cancer cell lines; the data is shown in Figure 6 and Table S4. POM-ACE showed mild anti-proliferative activity, where IC_50_ = 113 µg/mL. The strongest anti-proliferative activity (IC_50_ of 12–17 μg/mL) was observed for fractions 2F, 2G and 2K, which contain primarily UA, OA and their esters at varying concentrations.

**Figure 7:**
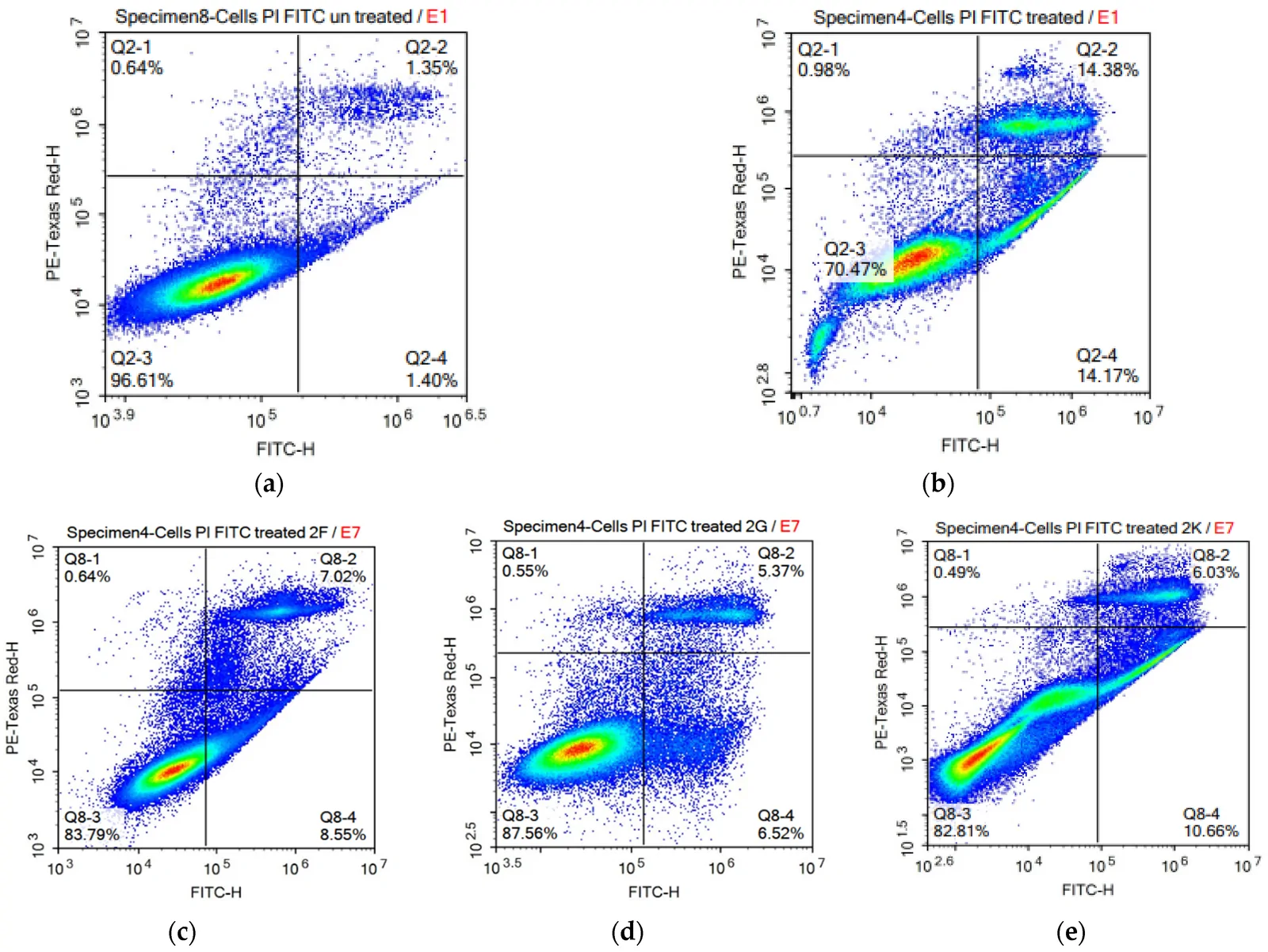
Dot plots of Annexin V/FITC from flow cytometry in HT-29 cells for (**a**) untreated control after 24 h (**upper left**), and (**b**) after 24 h treatment with UA (**upper right**); (**c**) fraction 2F (**lower left**), (**d**) fraction 2G (**lower middle**), or (**e**) fraction 2K (**lower right**).

The most effective fractions based on MTT assay data (2F, 2G, and 2K) were then evaluated by flow cytometry for the ability to induce cell death via apoptosis or necrosis, using the Annexin V-FITC/PI assay (Figure 7). Cells were treated for 24 h at the measured IC_50_ concentrations with each fraction. The major constituent of these fractions, UA, was also tested for comparison. UA induced both early (14.1%) and late apoptosis (14.3%). The fractions also induced both early and late apoptosis; relative percentage varied between treatments, where 2G induced 6.52% early and 5.37% late apoptosis, 2F induced 8.55% early and 7.02% late apoptosis, and 2K induced 10.6% early and 6.03% late apoptosis, when exposing cells at the approximate IC_50_ concentration.

### 2.3. Multivariate Analysis of Phytochemical Profiles with Anti-Proliferative Activity

**Figure 8:**
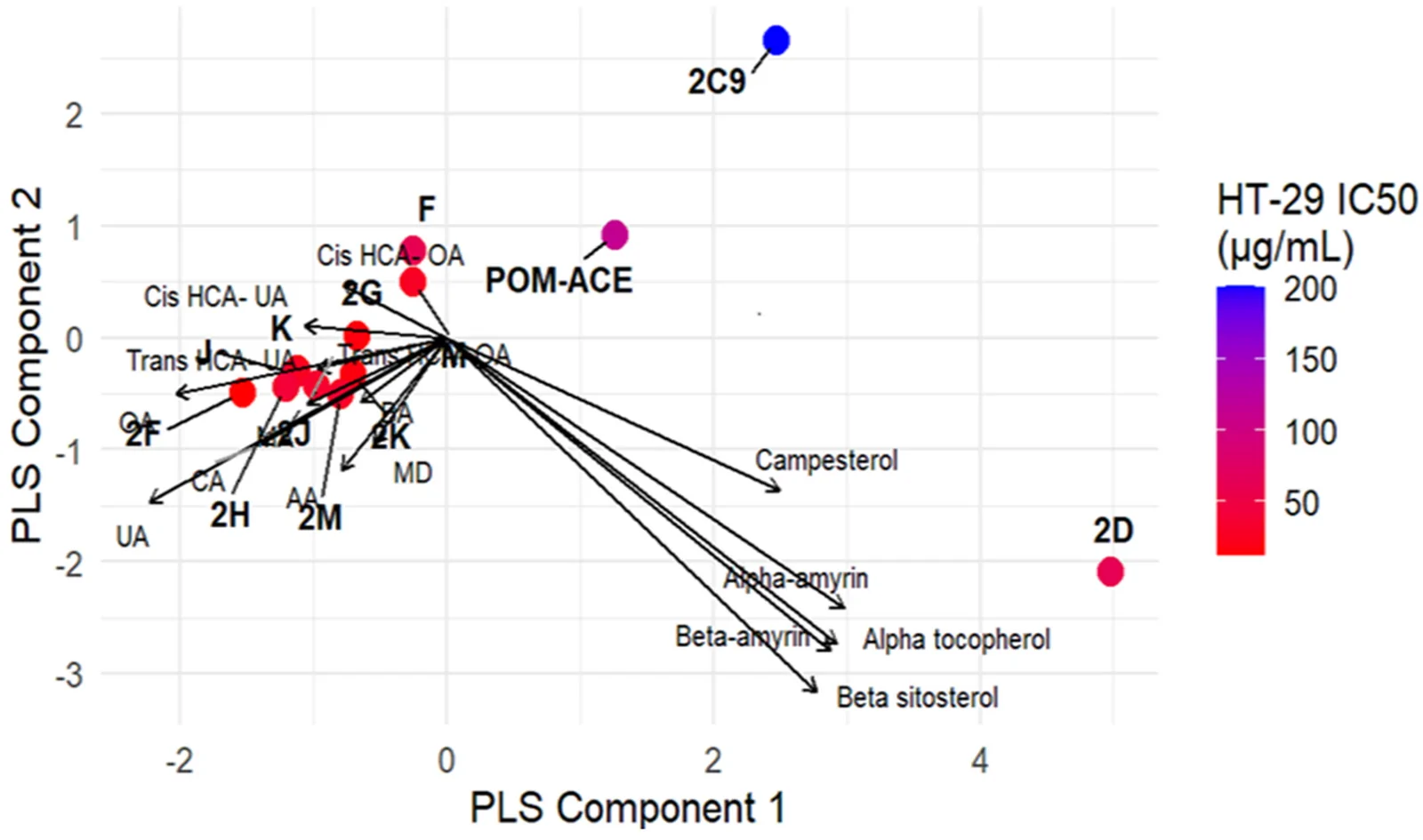
PLS biplot representing the content of major triterpenoids, sterols and tocopherols in POM-ACE and its fractions, vs. their anti-proliferative activity in HT-29 cells as the mean IC_50_ (*n* = 3).

Partial Least Squares (PLS) regression was used to analyze the correlation between the compositions of POM-ACE and its fractions, and their anti-proliferative activity (IC_50_, µg/mL) against HT-29 cells. A PLS biplot (Figure 8) shows the treatments color-coded by IC_50_, with red for higher activity (lower IC_50_) and blue for lower activity (higher IC_50_). Fractions with high activity, like 2F, 2G, and 2K, were clustered along the first latent variable (PLS Component 1), indicating shared chemical features linked to strong anti-proliferative effects. Less active samples, such as 2C9 and POM-ACE, were positioned away from the high-activity group, exhibiting distinct phytochemical profiles. The second PLS component further differentiates fractions with similar bioactivity, highlighting secondary differences in their composition.

### 2.4. Molecular Docking Data

**Figure 9:**
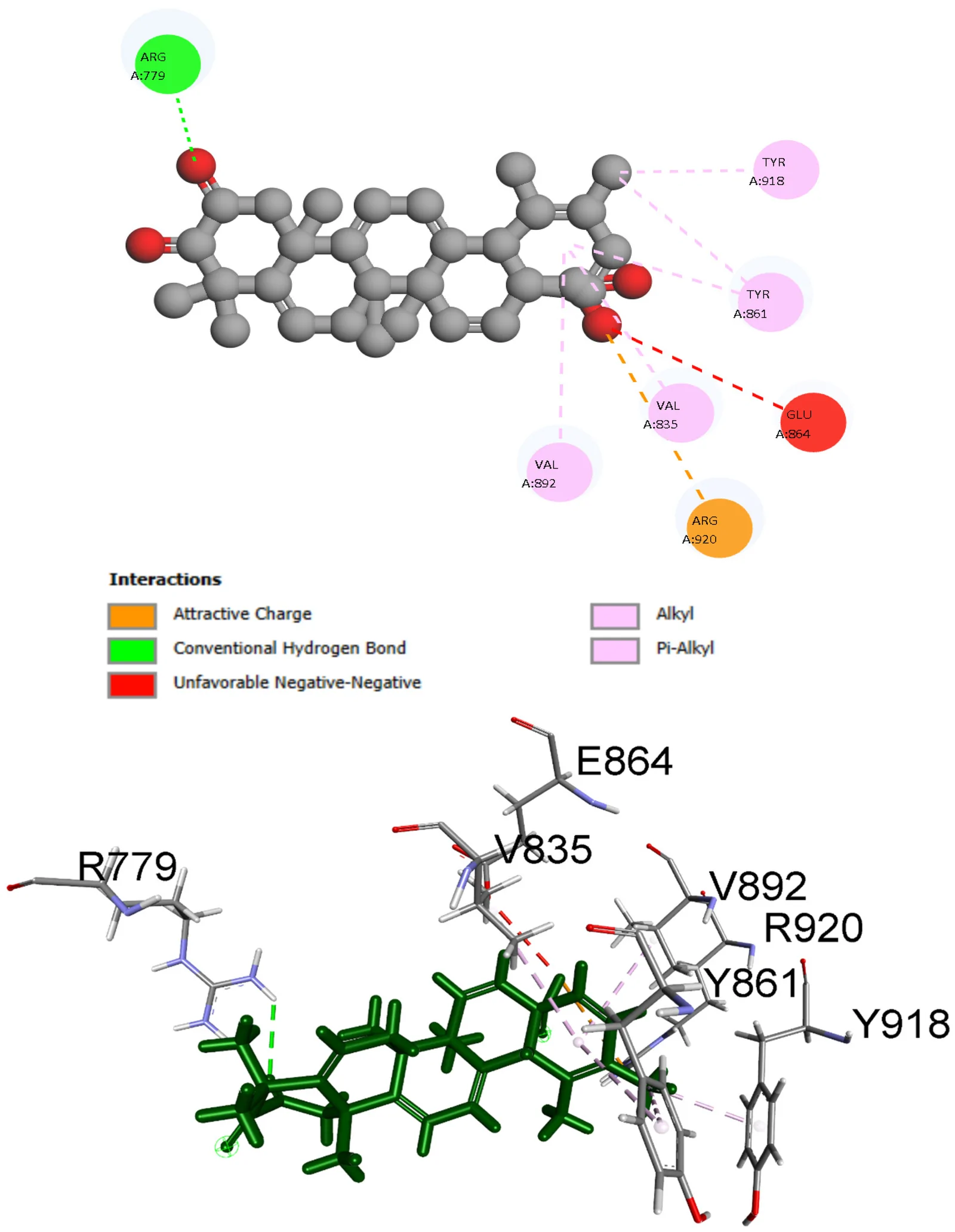
Molecular docking interactions for corosolic acid (CA) with NLRP3 inflammasome protein. Protein–ligand interactions, residues and intermolecular distances were visualized using BIOVIA Discovery Studio 2021 Client. All interaction types are clarified in Table S6.

In silico molecular docking with YASARA was used to evaluate potential interactions between the key constituents of pomace fractions (UA, OA, CA, α-amyrin, β-sitosterol, and α-tocopherol) that were likely to contribute to their bioactivities, and enzymes that play an important role in apoptosis (caspase-3 and caspase-9). We also examined possible interactions between these constituents and NLRP3 inflammasome protein. The interaction scores are summarized in Table 5. An example is shown for NLRP3 and corosolic acid (Figure 9). Data summarizing the residues involved and the type of interactions appears in the Supplementary Materials, Tables S5–S22.

## 3. Discussion

The goal of our study was to evaluate cranberry pomace as a source of triterpenoids and sterols and examine the potential contributions of these specific terpenoid constituents towards the inhibition of inflammation and colon carcinogenesis previously observed for cranberry. Towards this end, 100% acetone was used as an extraction solvent, as previous reports have shown it to be more effective for triterpenoids than methanol or ethanol [27]. Replacing methanol with 100% acetone also minimized the extraction of polyphenols, which are abundant in the pomace but outside the focus of this investigation. Ultrasound technology was used to synergize the solvent attack to the core of the complex structure of skin and seeds and effectively separate these nonpolar constituents from the pomace; ultrasonication for 30 min at room temperature optimized mass transfer into the solvent while avoiding degradation. Solute-to-solvent ratios were varied until optimal triterpenoid extraction was reached; a ratio of 1:10 minimized solvent consumption without sacrificing efficacy. From 100 g of freeze-dried pomace, 12.7 g of extract (POM-ACE) with a total triterpenoid content of 510 mg/g and minimal polyphenol was produced.

### 3.1. Fractionation and Analysis of POM-ACE

LCMS, GCMS and TLC analyses found POM-ACE to be a mixture containing multiple classes of biologically active terpenoids and other nonpolar metabolites, including triterpenoid acids, neutral triterpenoids, sterols, tocopherols and tocotrienols, carotenoids, waxes, and fatty acids. Effective fractionation was crucial to determine the contributions of these compounds towards biological activities, beginning with 100% hexane to isolate fatty acids and waxes and increasing polarity through stepwise increase in % ethyl acetate. Tocopherols, phytosterols, neutral triterpenoids and triterpenoid acids and esters were successively eluted, with polyphenols retained in later fractions. As polyphenols are also likely to possess anti-proliferative and anti-inflammatory properties, effective separation ensures we are assessing their contributions independently.

We found UPLC-MS reliable for the determination of triterpenoid acids and their HCA esters; however, our instrument’s electrospray ionization (ESI) system was less efficient for the ionization of nonpolar sterols, neutral triterpenoids and tocopherols. A reliable GC-MS method was developed and applied to identify and quantify neutral triterpenoids alpha-amyrin, beta-amyrin, lupeol, alpha-tocopherol, campesterol, beta-sitosterol, and stigmasterol in our fractions (Table 3). Using this method, cuticular waxes Triacontanal and Tritriacontane-12,14-dione (previously reported in blueberry [28]) were detected in some early eluting fractions (C, 2C1, 2C6, 2C9); however, these waxes show little to no activity in our assays compared to fractions D-M and 2D–2M, which contain primarily triterpenoids and sterols.

### 3.2. Effects of Cranberry Pomace Isolates in Cellular Models of Cancer and Inflammation

Previous studies by our group identified UA and its hydroxycinnamoyl esters from whole cranberry fruit as capable of inhibiting the growth of numerous cancer cell lines [12,13]. We further demonstrated that a nonpolar extract of whole cranberry fruit, rich in UA and beta-sitosterol, could inhibit colonic inflammation and tumorigenesis in a mouse model of colitis and downregulate the tissue expression of inflammatory cytokines IL-6, IL-1b and TNF-a [26]. Our previous study on triterpenoid content in cranberry raw materials and products identified cranberry pomace as an abundant source of free UA and OA and their HCA esters, with lesser quantities of CA and MA [3]. As these compounds are concentrated in the peel and have low water solubility, most are retained in the pomace when fruit is processed in the production of cranberry juices and juice-derived products. They make up an estimated 6–7% of the pomace by mass. Thus, cranberry pomace was of interest for further studies.

POM-ACE extract demonstrated a moderate anti-proliferative effect on HT-29 colon adenocarcinoma cells as measured using MTT assay, with an IC_50_ of 113 µg/mL after 24 h of treatment. POM-ACE at 100 µg/mL also significantly inhibited LPS-induced expression of IL-1β in THP-1 monocytes, reducing levels by about 30%. This finding is consistent with our previous report showing that cranberry extracts rich in polyphenols or triterpenoid acids downregulated the production of IL-6, IL-1β and TNF-α in THP-1 monocytes [16] and illustrates the anti-inflammatory potential of cranberry triterpenoids.

Data from the THP-1 monocyte model on fractions C–M suggest possible NLRP3 inflammasome modulation (Figure 5). Fractions rich in neutral triterpenoids and sitosterol (D), or HCA esters of UA and OA (J) significantly reduced IL-1β expression at microgram concentrations in THP-1 monocyte cells. Fractions K and M show similar dose-dependent inhibition of IL-1β expression at higher concentrations (50–100 ppm); these contain about 5% CA in addition to UA and OA. Analysis of the active fractions revealed the major constituents of fraction D to be alpha-amyrin, beta-amyrin, and beta-sitosterol, accounting for approximately 80% of D by mass. Wu and coworkers recently demonstrated that β-sitosterol produced an inhibitory effect on the NLRP3 inflammasome and reduced IL-1β production in a rat model of myocardial injury [29]. Fraction J contained over 40% HCA esters of UA and OA, which may account for its improved efficacy compared to K and M. Through molecular docking analysis, we confirmed favorable interaction scores (Table 5) for NLRP3 with UA, OA, CA, α-amyrin and β-sitosterol, suggesting that ligand–protein complex formation is possible. Among these, CA exhibited the best interaction score (8.5 kcal/mol), interacting with eight different residues through hydrophobic, hydrogen bonding and electrostatic interactions as depicted in Figure 9. By comparison, alpha-tocopherol showed a weaker interaction with NLRP3 (5.6 kcal/mol).

Though present at a low concentration in D, tocopherol may not contribute as strongly to inflammasome inhibition as the amyrins or sitosterol. Fraction C, which contained only trace triterpenoids and 2 mg/g tocopherols, exhibited limited activity against cytokine inhibition in the THP-1 monocyte model. According to a report by Angsusing and coworkers, β-amyrin and stigmasterol, the major constituents in yataprasen formulation, displayed strong anti-inflammatory activity by suppressing LPS-induced IL-1β, IL-6, and TNF-α production in THP-1 cells [30]. Thus, our findings are consistent with known activities of amyrins and sitosterol.

A limitation of the present study is that THP-1 cell viability was not evaluated at the same fraction concentrations, and exposure time used for the IL-1β assay. Consequently, a contribution of fraction-induced cytotoxicity to the reduction in IL-1β secretion cannot be fully excluded. Nevertheless, the fraction- and concentration-dependent responses observed in the THP-1 model support the anti-inflammatory potential of the active fractions. Further studies should include matched cell-viability assays and the direct evaluation of inflammasome-associated endpoints, including NLRP3 activation, caspase-1 activation, gasdermin D cleavage, and mature IL-1β release, to confirm the specificity and molecular basis of the observed effects.

Lipoxygenase (15-LOX) is an important enzyme that catalyzes the oxidation of polyunsaturated fatty acids, such as arachidonic acid, and contributes to inflammatory processes by generating pro-inflammatory lipid mediators. For this reason, the selected POM-ACE fractions were evaluated for their inhibitory effects on 15-LOX activity, as outlined in Section 4.6. The LOX inhibition data (Table 4) indicate that 2G and 2H fractions were the most effective enzyme inhibitors. When tested alone, triterpenoid acids UA and CA were less potent LOX inhibitors than fractions containing mixtures of triterpenoids. The 2G and 2H fractions exhibited stronger LOX inhibitory activity than pure UA and CA, suggesting a possible synergistic effect among multiple constituents—such as triterpenoid acids and their ester derivatives—present within these fractions. As discussed above, Fraction 2G contains a mixture of UA and OA, making up over 70% of the fraction, while the remainder (approximately 29%) is made up of the four p-hydroxycinnamoyl esters of UA and OA. Fraction 2H contains about 93% triterpenoid constituents, with ursolic and OA making up over 80% of the fraction, and CA and MA making up about 8.3%. In both 2G and 2H, UA is the predominant triterpenoid component. UA and OA have both been previously reported as inhibitors of soybean-derived 15-lipoxygenase [31]; our data is consistent with this report. Moreover, it suggests that the combination of different triterpenoids in these fractions could produce a synergistic effect on LOX inhibition; for example, the hydroxycinnamoyl esters may function as strong LOX inhibitors, enhancing the effects of UA.

MTT assay data (Figure 6) indicated that the relative anti-proliferative effects on HT-29 cells for many fractions were stronger than that of the parent extract. Treatment with POM-ACE extract produced moderate inhibition of HT-29 colon cell adenocarcinoma proliferation over 24 h based on MTT assay data (IC_50_ = 113 µg/mL). While the extract contains over 50% triterpenoids by weight, it also contains significant quantities of other nonpolar constituents, including carotenoids, phytosterols, tocopherols, and long-chain fatty aldehyde tricontanal, which are sparingly soluble in PBS or cell culture media. Reduced solubility or activity of these components may decrease the overall cytotoxicity of POM-ACE. Triterpenoid-rich fractions of POM-ACE showed moderate anti-proliferative activity against HT-29 cancer cell lines, while fractions low in triterpenoids and high in nonpolar constituents such as waxes were much less effective. Beta-sitosterol alone was a more effective inhibitor of HT-29 proliferation than sitosterol-rich fraction 2D. Similarly, UA alone (IC_50_ = 4.4 µg/mL) was more effective than the UA-rich fractions (IC_50_ ranged from 12 to 39 µg/mL). An IC_50_ of 11.9 µg/mL (26 µM) in HT-29 cells was previously reported for UA [19], which is consistent with our findings. Although not as effective as anti-cancer drugs such as doxorubicin (reported IC_50_ = 1.35 µg/mL or 2.5 µM in HT-29 cells [32]), these extracts demonstrate potential utility. Our data suggests that among the nonpolar constituents of cranberry pomace, UA and OA and their HCA esters are all likely to contribute to the reduced colon cell proliferation. Fractions 2F, 2G, and 2K, which were the most effective in reducing HT-29 proliferation, contain approximately 86%, 99%, and 76% of these triterpenoids, respectively.

Multivariate analysis was used to correlate chemical composition with the inhibition of HT-29 colon cancer cell proliferation. Analysis of PLS loadings (Figure 8) revealed that UA, OA, and their HCA esters significantly contributed to the PLS model. They clustered with highly active fractions, indicating their key role in HT-29 cytotoxicity. Phytosterols and amyrins loaded in different directions from the acids and esters, corresponding to samples with weak or moderate activity. The strong correlation between UA and OA and their potent inhibition of HT-29 cells aligns with established anti-cancer mechanisms of pentacyclic triterpenoids. These include inducing mitochondrial apoptosis, increasing oxidative stress, activating caspases, and modulating NF-κB and PI3K/Akt signaling pathways [18,33,34,35,36,37,38,39,40]. We have previously reported that *cis*- and *trans*-hydroxycinnamoyl UA isolated from cranberry fruit can inhibit the growth of multiple cancer cell lines, including HT-29 [13].

Flow cytometry experiments in HT-29 cells indicate that triterpenoid constituents of POM-ACE and its fractions are capable of inducing early and late-state apoptosis. Treatment with fractions 2F, 2G and 2K at near IC_50_ concentrations over 24 h (Figure 7) resulted in apoptosis for approximately 12–16% of HT-29 cells. UA alone resulted in approximately a 14% increase in early or late-stage apoptosis, which is consistent with levels previously reported for UA in HT-29 cells at 20 µM [19]. Previous studies reported that UA can induce apoptosis in part by promoting cleavage of the procaspases and increasing the activation and release of caspase-3 and -9 [19,41]. It is not known whether triterpenoids like those in POM-ACE can directly interact with these caspases. To explore the possibility that such interactions could be energetically favorable, we investigated the binding affinities of triterpenoids and other constituents toward caspase-3 (executioner caspase) and caspase-9 (initiator caspase), which could drive conformational changes and support apoptosis. In silico molecular docking analysis with YASARA indicates that several triterpenoid constituents interact favorably with caspase-3 and -9 (Table 5). The major constituent of most of the active fractions, UA, interacts with caspase-3 (8.6 kcal/mol) and caspase-9 (7.8 kcal/mol); another major constituent, OA, also demonstrates favorable interactions with caspase-3 (interaction score 9.1 kcal/mol) and caspase-9 (interaction score 7.9 kcal/mol). Our results are consistent with previous in silico studies which indicated favorable interactions for OA with caspase-3 [42]. Results are also consistent with a previous report showing that UA could induce apoptosis in HT-29 cells through the activation of caspase-3 and caspase-9 [19]. β-sitosterol also demonstrated strong interactions with caspase-3 and caspase-9. Among the minor constituents of pomace, both α-amyrin and CA demonstrate favorable interactions with caspase-3 and -9. The data herein supports a possible role for the major triterpenoid constituents of fractions 2F, 2G, and 2K in reducing HT-29 cell proliferation through apoptosis. Further studies would be needed to confirm whether fractions derived from POM-ACE can promote the cleavage of caspase-3 and -9, as well as to investigate their effects on other pathways associated with reducing colon cancer cell proliferation.

### 3.3. Potential Benefits of Cranberry Pomace Terpenoids

As an abundant source of triterpenoids, cranberry has the potential to protect against a variety of inflammation-linked conditions including various cancers. Published work on specific triterpenoids indicates that UA can be beneficial in reducing inflammation in osteoporosis, arthritis, intestinal diseases, and others. In addition, it has been shown to induce apoptosis in cancer cells such as human breast cells, osteosarcoma cells, cervical adenocarcinoma cells, and colorectal cancer cells [34]. Research on OA and its derivatives shows its anti-inflammatory effects occur through regulating the expression of pro-inflammatory cytokines (IL-1β, IL-6, IL-12, TNF-α) and lipid mediators (COX-1, COX-2) [18]. OA was found to decrease the proliferation of HCT-15, HT-29, HCT-8, and Colo 205 human colon cancer cells in a dose-dependent manner [40]. Likewise, the less-plentiful triterpenoids in cranberry pomace may be expected to contribute to its anti-inflammatory and anti-cancer properties. MA, a minor cranberry triterpenoid, reportedly inhibits cell proliferation of the HT-29 human colon cancer cell line by inducing apoptosis [43]. MA has demonstrated potential inhibitory effects against multiple cancers, including colorectal, pancreatic, lung, leukemia, prostate, bladder, gallbladder, esophageal, and gastric cancers. In addition, studies have reported that MA can play a role in reducing neuroinflammation [44] as well as mitigating cardiovascular disease, asthma, neurodegenerative disorders, diabetes, and obesity [45]. CA exerts anti-cancer and anti-inflammatory activities against liver cancer, gastric cancer, colon cancer, and lung cancer [46] breast cancer [47], and non-alcoholic steatohepatitis [48]. BA possesses anti-inflammatory and anti-cancer activities against ulcerative colitis, rheumatoid arthritis, and acute pancreatitis [49], brain tumors, and various cancers including breast, cervical, colorectal, endometrial, gallbladder, gastric, lung, hepatocellular, leukemia, lymphoma, melanoma, oral, ovarian, pancreatic, prostate, and renal cancer [50].

β-sitosterol and other sterols found in cranberry pomace are also likely to contribute to anti-cancer properties. Previous studies of sitosterol’s anti-proliferative properties include leukemia, lung cancer, stomach cancer, breast cancer, colon cancer, ovarian cancer, and prostate cancer [51]. β-sitosterol can avert neutrophil migration to the lateral line and diminish the expression of inflammatory genes (il-8 and myd88) to suppress inflammation [52]. Sitosterol [53] and campesterol [54], also detected in pomace, may be beneficial for rheumatoid arthritis. Several pomace fractions (2H, 2J, 2K, and 2M) contained trace quantities of asiatic acid and madecassic acid. Asiatic acid possesses anti-cancer and anti-inflammatory activities on colon cancer [55], while madecassic acid may eradicate colitis-associated colorectal cancer through inhibition of IL-17 expression [56]. Inhibition of lung cancer [57] and leukemia [58] are also reported. Other trace constituents of pomace with potential cancer-inhibitory properties include tocopherols and tocotrienols, which likely originate from the seeds. Tocotrienol, which has been reported for anti-breast cancer properties, is present in 2C9 [59]. Several pomace fractions (C, 2C1, 2C6, 2C9) contained <1% alpha-tocopherol, D and 2D contained 1.7% and 3.1% respectively. Alpha-tocopherol is an important antioxidant associated with the prevention of atherosclerosis and the reduction of cardiovascular disease [60].

The combination of triterpenoid acids and esters, sterols, and tocopherols makes cranberry pomace an excellent source of bioactives with the potential to improve colon health through a variety of complementary pathways including inhibition of inflammasome activation, LOX activity and abnormal cell proliferation.

## 4. Materials and Methods

### 4.1. Plant Materials and Reagents

Cranberry pomace, a labeled solid sample, was collected from Ocean Spray Cranberries Inc. (Lakeville, MA, USA). All cranberry pomace was stored at −20 °C until use. Reagent-grade acetone was purchased from Pharmco by Greenfield Global (Brookfield, CT, USA); UV/HPLC/LCMS grade acetonitrile, methanol, and water from Fisher Chemical (Pittsburgh, PA, USA). The HPLC grade for phosphoric acid was purchased from Sigma Aldrich (St. Louis, MO, USA). Formic acid (LC/MS grade) was purchased from Fisher Chemicals. Ursolic acid, oleanolic acid, betulinic acid, maslinic acid, and corosolic acid were obtained from Extrasynthese (Genay, France). Beta-sitosterol was purchased from Indofine Chemical Company Inc. (Hillsborough, NJ, USA). Madecassic acid, and asiatic acid were purchased from Cayman Chemical. Alpha-tocopherol and Gamma-tocopherol were purchased from Millipore Sigma, Burlington, MA, USA. Alpha-amyrin with a purity of ≥98.5% was purchased from Extrasynthese (Genay, France). Further, 20% phosphomolybdic acid was purchased from Sigma Aldrich, St. Louis, MO, USA. Silica gel 230–400 mesh was purchased from Natland International Corporation, Morrisville, NC, USA. Gibco fetal bovine serum (FBS), 0.25% Trypsin-EDTA, and antibiotic antimycotic were purchased from Thermo Fisher Scientific (Waltham, MA, USA). McCoy’s 5A medium was purchased from Sigma Aldrich (St. Louis, MO, USA). HT-29 Human colon cancer cells were purchased from the American Type Culture Collection (ATCC; Manassas, VA, USA). Human IL-1β ELISA MAX Deluxe kit was purchased from BioLegend (San Diego, CA, USA). Roswell Park Memorial Institute (RPMI) 1640 medium, Phorbol myristate acetate (PMA), lipopolysaccharide (LPS), and THP-1 monocyte cells were purchased from ATCC (Rockville, MD, USA). Lipoxygenase inhibitor screening assay kit (item: 760700) was purchased from Cayman Chemical Company (Ann Arbor, MI, USA). MTT (3-(4,5-dimethylthiazol-2-yl)-2,5-diphenyltetrazolium bromide; lot number 4093092) was purchased from EMD Millipore Corp (Burlington, MA, USA).

### 4.2. Extract Preparation

A total of 600 g of frozen cranberry pomace was thawed and ground to a fine powder using a coffee grinder. The pomace powder was freeze-dried (Labconco Freezone 4.5 L, Labconco, Kansas City, MO, USA) at −85 °C for 48 h to yield approximately 200 g of dried pomace powder. In total, 100 g cranberry pomace powder was suspended in 1000 mL reagent-grade acetone. Ultrasonication was performed for 30 min with occasional shaking using an ultrasonicator (Fisher Scientific FS20H, Fisher Scientific, Hampton, NH, USA) at room temperature, after which the suspension was vacuum-filtered using P8 filter paper. The solid residues were reextracted twice for 30 min each time using 500 mL acetone. The filtrates were combined and concentrated by a rotary evaporator (Buchi Labortechnik AG, Flawil, Switzerland), then freeze-dried at −85 °C to produce a solid extract, POM-ACE (12.7 g).

### 4.3. Fractionation of POM-ACE Extract

To produce semi-purified fractions concentrated in specific secondary metabolites, column chromatography was performed on silica gel (230–400 mesh), starting with 1 g and 5 g batches, respectively. For Fractionation #1: a (50 × 3 cm) column was packed with 53 g silica gel 60, mesh size 230–400. A total of 1.005 g of POM-ACE was suspended in 30 mL n-hexane with ultrasonication and vortexing to assist dissolving, and was loaded carefully on the column. The column was eluted with mixtures of n-hexane/ethyl acetate. Beginning with 100% n-hexane to elute oily components, including fatty acids and carotenoids, then sequentially increasing the solvent system polarity by increasing ethyl acetate up to 50:50 hexane/ethyl acetate to elute most of the sterols and triterpenoids. Fractions were pooled according to qualitative analysis by thin-layer chromatography and renamed as fractions A–M, as indicated in Table S1.

Fractionation #2: A (50 × 5 cm) glass column was packed with 150 g silica gel (mesh size 230–400), packed height = 22 cm. A total of 5.004 g of POM-ACE extract was suspended in 100 mL 100% n-hexane with ultrasonication and vortex to aid in dissolving, then applied to the column. Fractions were eluted using a similar solvent system as Fractionation #1. Initially, 100% n-hexane was selected to elute fatty acids, carotenoids, and other oily components, then, the solvent system polarity was sequentially increased by increasing ethyl acetate (up to 50:50 hexane/ethyl acetate) to elute most of the sterols and triterpenoids. Finally, the polarity of the mobile phase was increased by using up to 50% methanol to elute remaining secondary metabolites. This set of fractions is named 2A–2M according to Table S2, which also summarizes solvent systems used for chromatography, total fraction weights after pooling and results of qualitative TLC analysis of the major fractions.

### 4.4. Quantitative Analysis of Triterpenoids in Cranberry Pomace Extracts by UPLC-MS

UPLC-MS was used to determine UA, OA, CA, MA, betulinic acid (BA), *cis*- and *trans*-3-O-p-hydroxycinnamoyl-UA and -OA, and other triterpenoid acids on Waters Xevo QTOF MS and ACQUITY UPLC instrumentation using a modification of a published method [3]. Briefly, POM-ACE extracts and fractions (1–1000 ppm in methanol) were analyzed on a Waters Acquity UPLC BEH C18 column (100 × 2.1 mm, 1.7 µm) with isocratic elution (85% methanol + 0.1% formic acid, 15% water + 0.1% formic acid) at 0.3 mL/min for 20 min. Injection volumes (1–3 µL) were made by an autosampler. Negative ESI-MS full-scan mode (*m*/*z* 100–1000) was used under optimized conditions (capillary voltage: 2 kV, cone voltage: 40 V, source temperature: 100 °C, desolvation temperature: 350 °C, cone gas flow (L/h): 50, desolvation gas flow (L/h): 600). Madecassic acid and isomers were detected at *m*/*z* of 503.3, and asiatic acid and isomers at *m*/*z* 487.3. UA, OA, and BA were monitored at *m*/*z* of 455.4, CA and MA were detected at *m*/*z* of 471.4, and *cis*- and *trans*-3-*O*-p-hydroxycinnamoyl UA and OA were analyzed at *m*/*z* of 601.4. Identification of triterpenoids was accomplished by matching the retention times and *m*/*z* with the commercial authentic standards (UA, OA, CA, MA, BA) and previously published data [3], and quantitation was performed using the external standard curve method. Samples were analyzed in triplicate. Metabolite content is reported as the mean +/− the standard deviation. Data analyses were performed using MassLynx software version 4.1.

### 4.5. Quantitative Analysis of Phytosterols, Tocopherol, and Neutral Triterpenoids by GC-MS

Quantitative analysis of beta-sitosterol, tocopherol, and neutral triterpenoids was carried out on a Thermo Focus ITQ GC-MS system, using a published method with minor modifications [61]. A TG-SQC GC column (30 m × 0.25 mm, i.d., 0.25 µm film thickness) nonpolar column was used as the stationary phase, supplied by Thermo Scientific. Helium gas was used as a mobile phase or carrier gas at a constant flow rate of 1 mL/min. A quadrupole ion trap (ITQ 700) was used as a mass analyzer (Thermo Fisher Scientific, Austin, TX, USA). The oven temperature was initially set at 60 °C, programmed to 300 °C at a rate of 40 °C per minute, and held for 14 min with a solvent delay of 6 min. The injection volume is 1 μL, with the spitless mode at 1 min. The inlet temperature was set to 300 °C. The ionization mode was positive, with electron ionization mode at 70 EV. Mass transfer or interface temperature was set at 280 °C. The ion source temperature was set at 230 °C. The scanning type was full-scan mode. Mass range (*m*/*z*) was 50–650 Da. α-amyrin, β-amyrin, and lupeol were monitored at *m*/*z* of 426.16, and the base peak was 218.07. β-sitosterol was monitored at *m*/*z* of 414.14. Campesterol was monitored at *m*/*z* of 400.1. α-tocopherol was monitored at *m*/*z* of 430.2. β-tocotrienol was monitored at *m*/*z* of 410.1. Compounds were identified by matching the retention times and *m*/*z* with the commercial authentic standards (β-sitosterol, campesterol, α-amyrin, α-tocopherol) in the NIST Mass Spectral Library. Samples were analyzed in triplicate. Data analysis was performed using Xcaliber software version 2.1. Quantification results in comparison to standards are reported as the mean +/− the standard deviation.

### 4.6. Inhibition of Lipoxygenase (LOX) Activity

The inhibitory effects of POM-ACE fractions on LOX activity were assessed using the LOX enzyme kit according to the manufacturer’s protocol [62]. A 96-well plate contained blank wells with 90 μL of assay buffer and 10 μL of DMSO, control wells containing 90 μL of 15-Lipoxygenase and 10 μL of DMSO, and the remaining wells contained the test samples (inhibitor) containing 90 μL of lipoxygenase enzyme and 10 μL of each test sample (5–500 µg/mL). Incubation was performed for five minutes at room temperature. The reaction was initiated by adding 10 μL of the substrate arachidonic acid to all wells, then the 96-well plate was placed on a shaker. After ten minutes, 100 μL of chromogen was added to each well to stop enzyme catalysis and develop the reaction. The plate was covered and placed on a shaker for another five minutes, then the cover was removed, and the absorbance was read at 490 nm using a SpectraMax M2 plate reader (Molecular Devices; Sunnyvale, CA, USA). Percent inhibition of LOX activity for each sample was calculated as:

%Inhibition = (Absorbance Control − Absorbance Sample) × 100/Absorbance Control

Approximate IC_50_ for selected fractions, POM-ACE, and standards CA and UA were calculated using Probit analysis software by LdP Line (EhabSoft, Cairo, Egypt) which calculates the IC_50_ value and generates a concentration–response curve.

### 4.7. Evaluation of Anti-Inflammasome Effects in THP-1 Monocyte Model

The anti-inflammasome activity of pomace extract and fractions was evaluated by measuring the LPS- and nigericin-induced cytokines according to a reported method [63]. THP-1 monocytes were seeded at a density of 50,000 cells per well in a 48-well plate using Roswell Park Memorial Institute (RPMI) 1640 medium supplemented with 10% fetal bovine serum with PMA (50 ng/mL) at 37 °C, with 5% humidified CO_2_, and differentiated for 48 h. Then, the culture medium was removed and replaced with PMA-free medium for another 24 h. LPS (100 ng/mL) was added for 4 h to induce inflammation, followed by the addition of POM-ACE and selected fractions at concentrations ranging from 1.25 to 100 µg/mL for 1 h. Then, nigericin (10 μM) was added for 1 h. Supernatants were collected, and an ELISA kit was used to measure the effect of treatments on pro-inflammatory cytokine production (IL-1β). MCC950, a known inflammasome inhibitor, is the positive control. A natural product, resveratrol, is also used as a reference compound. Statistical analysis employing one-way ANOVA with multiple comparisons was conducted using GraphPad Prism 8, as previously described [16].

### 4.8. Evaluation of Anti-Proliferative Effects on HT-29 Colon Adenocarcinoma Cells

The human colon cancer HT-29 cell line was cultured according to ATCC’s recommendations at 37 °C and 5% CO_2_ in McCoy’s 5A medium, modified and supplemented with 10% fetal bovine serum, 10,000 units/mL of penicillin, 10,000 μg/mL of streptomycin, and 25 μg/mL of Amphotericin B. Cells were trypsinized with 0.25% trypsin-EDTA and sub-cultured in the same media at 72 h intervals. To determine the effects of pomace extract and fractions on cell viability and proliferation, the MTT [3-(4,5-dimethylthiazol-2-yl)-2,5-diphenyltetrazolium bromide] colorimetric assay was conducted in 96-well plates using a published procedure [64] with minor modifications. Briefly, HT-29 cells were seeded in 96-well plates at a density of 10,000 cells/well. Stock solutions of test samples and standards were initially prepared in DMSO and media by vortexing and sonication, then diluted with media to the appropriate test concentrations (final DMSO concentrations in the wells of 0.1% or less). Following a 24 h incubation, the cells were treated with test samples and standards (1.5–300 µg/mL) for 24 h. Following incubation, the media was replaced with serum-free media, and 10 µL of MTT solution was added. Cells were incubated with MTT for 2 h at 37 °C with 5% CO_2_ to induce formazan crystal formation. DMSO was added to solubilize the formazan crystals and plates placed on a Thermo Scientific MAXQ 4450 benchtop orbital shaker, model SHKA4450 (Thermo Fisher Scientific, Waltham, MA, USA), at 37 °C for 30 min, after which the absorbance was measured using a SpectraMax i3 plate reader (Molecular Devices, Sunnyvale, CA, USA) with SoftMax Pro 6.3 version software, at a wavelength of 570 nm, and compared to untreated control. The cytotoxic potential is calculated using the following formula:

% Growth inhibition = 100 − (Sample treated cells abs − blank abs)/(Untreated cells abs − blank abs) × 100.

IC_50_ values (50% inhibition of cell growth) were calculated using Probit analysis (LdP Line software, https://www.ehabsoft.com/ldpline/). Data are presented as the mean ± standard deviation (SD), calculated using Microsoft Excel.

### 4.9. Evaluation of Apoptosis in HT-29 Cells Using Flow Cytometry

Test samples of 2F, 2G, 2K and UA were prepared, as described in the previous section, at concentrations near the measured IC_50_. Cells were seeded at a density of 2 × 10^5^ cells per well in a 6-well plate for 24 h. Subsequently, the seeded cells were treated with a near-IC_50_ concentration for 24 h (18.7 ppm for each fraction, 6.25 ppm for UA). A total of 400 µL of trypsin-EDTA was used for 5 min to detach adherent cells, thereby avoiding prolonged trypsinization and preventing damage and false positives. Cell suspensions were centrifuged for 10 min at 1400 rpm to pellet the cells, and the supernatant was discarded. Cells were washed once with cold PBS and centrifuged for 10 min to pellet the cells. After discarding the supernatant, they were washed with 1× binding buffer and centrifuged for 10 min to pellet the cells. Pellets were resuspended in 1mL 1× Annexin V binding buffer at a concentration of 1 − 5 ×10^6^ cells/mL.

Staining with Annexin V and propidium iodine was performed using an eBioscience™ Annexin-V Apoptosis Detection Kit (Invitrogen™, Thermo Fisher Scientific, Waltham, MA, USA) according to the manufacturer’s instructions [65], with minor modifications. Briefly, 5 µL of Annexin V-FITC was added to 190 µL of the cell suspension in a new 96-well plate. Gently mix. Subsequently, 5 µL of Propidium Iodide (PI) staining solution was added, and the mixture was incubated at room temperature in the dark for 15 min, then analyzed by flow cytometry within 1 h. Finally, the cells were analyzed using a NovoCyte flow cytometer (Agilent Technologies, Inc., Santa Clara, CA, USA). Untreated cells, unstained treated cells, only annexin-V-stained cells, only PI-stained cells, and annexin-V- and PI-stained cells for automatic compensation on the flow cytometer.

### 4.10. Correlation of Bioactivity with Composition by Multivariate Analysis

Multivariate analysis using R (version 4.4.2) was used to visualize pairwise relationships between bioactive chemical compositions analyzed by LC-MS and GC-MS and pharmacological properties of lipoxygenase (LOX) inhibition and HT-29 anti-proliferative activity. Pearson correlation coefficients were used to assess the strength and direction of linear associations in the dataset. A multivariate Partial Least Squares (PLS) regression model was conducted to visualize chemical correlation with pharmacological effects [66]. This approach helped identify key compounds responsible for the biological effects observed in each bioassay. The software used was R version 4.4.2 (2024-10-31). AI-based language model ChatGPT Version 5 was used to assist in generating R scripts for multivariate statistical analysis. All codes were executed by the author using R, who performed data analysis and interpretation and conclusions.

### 4.11. Molecular Docking Studies

An in silico docking study of POM-ACE constituents and potential targets was conducted using YASARA software to probe protein–ligand interactions. The 3D structures of NLRP3 (AF-Q96P20-F1-model_v6), Caspase-1 (PDB ID: 1SC3), Caspase-3 (PDB ID: 1QX3), and Caspase-9 (PDB ID: 1JXQ) were downloaded from the RCSB PDB data bank and the UniProtKB [67,68]. To start the cleaning procedure of protein, natural ligands and water molecules were eliminated. Before performing energy minimization, the hydrogen atoms were added by YASARA software. The PDB format of the ligand was constructed using ChemSketch software (Version 2025). Finally, energy minimization was done by using YASARA software [69]. Molecular docking-based interaction analysis was conducted using the dock_run.mcr macro in YASARA software (version 22.8.22.W.64), where 25 AutoDock Vina (Version 22.8.22.W.64) docking runs were clustered to identify representative binding conformations [70]. The AMBER14 force field was applied using default parameters. Gibbs free energy (ΔG, kcal/mol) values were calculated within YASARA, with higher positive energy scores indicating stronger ligand–receptor interaction [71]. Docking poses were ranked using YASARA’s default energy-based scoring function. The top-ranked conformations were evaluated based on their proximity to key binding-site residues, binding energy, and ligand–protein interactions. The optimal binding pose was further refined through energy minimization using the AMBER force field. Docking outputs were saved in CSV format for statistical analysis and PDB format for structural visualization. Protein–ligand interaction residues, intermolecular distances, and bonding interactions were visualized with BIOVIA Discovery Studio 2021 Client [72].

## 5. Conclusions

Cranberry pomace contains a variety of triterpenoids and sterols with the potential to limit inflammation and the development of colon cancer. These compounds can be readily isolated using ultrasound-assisted extraction and quantified using UPLC-MS and GCMS methods. Bioassay-guided fractionation yielded extracts rich in UA, OA, and their esters, as well as other triterpenoid acids, neutral triterpenoids and phytosterols. The present study demonstrates that these compounds may act in a complementary manner to reduce inflammation through more than one route. Cytokine (IL-1β) inhibition was demonstrated in THP-1 monocytes for fractions rich in amyrins and β-sitosterol, and fractions rich in UA, OA, and their HCA esters. Molecular docking data demonstrate favorable binding interactions of NLRP3 protein with several of these compounds, which suggests that the observed IL-1β inhibition may be associated with inflammasome modulation; however, further experiments would be necessary to confirm this. The combination of UA, OA, and their trans-HCA-esters in specific fractions correlated positively with inhibition of lipoxygenase activity, indicating that cranberry-derived triterpenoids may also reduce inflammation by acting in a synergistic manner to target an important enzyme in arachidonic acid metabolism. Moreover, fractions rich in UA, OA, and their HCA esters also markedly reduced the proliferation of HT-29 colon adenocarcinoma cells, in part by inducing apoptosis, indicating potential for the major triterpenoids in pomace to inhibit tumor growth in addition to reducing inflammation. Ongoing studies will further examine mechanisms by which pomace-derived triterpenoids and sterols can reduce inflammation and inflammation-linked cancers. Our study demonstrates the potential of the unique combination of terpenoids found in cranberry pomace to protect against inflammation-linked conditions such as inflammatory bowel diseases and colon cancer.

## Figures and Tables

**Table 1:** Contents of major triterpenoid acids and esters detected by UPLC-MS in POM-ACE and fractions C–M from separation of 1 g POM-ACE (mg/g dry weight ± SD, *n* = 3).

Constituents	POM-ACE	C	D	F	J	K	M
UA	263 ± 3.0	0.78 ± 0.16	3.95 ± 0.04	336 ± 5	332 ± 6.6	487 ± 10	558 ± 6.6
OA	70.2 ± 1.3	0.34 ± 0.02	1.21 ± 0.02	179 ± 8	185 ± 2.3	185 ± 3.2	81.4 ± 2.1
CA	14.8 ± 0.7	ND	0.27 ± 0.0	ND	0.41 ± 0.02	56.8 ± 0.4	49.1 ± 1.0
MA	4.40 ± 0.25	ND	0.10 ± 0.0	ND	ND	28.2 ± 2.5	13.4 ± 0.2
BA	0.41 ± 0.01	ND	0.04 ± 0.0	0.05 ± 0.01	0.05 ± 0.01	ND	ND
*Cis*-HCA-OA	8.8 ± 1.3	ND	0.45 ± 0.02	38.7 ± 9.7	10.5 ± 0.23	0.45 ± 0.07	0.01 ± 0.007
*Cis*-HCA-UA	17.0 ± 9	ND	3.10 ± 0.17	203 ± 11	118 ± 10.8	7.46 ± 0.3	0.28 ± 0.003
*Trans*-HCA-OA	7.3 ± 1	ND	0.17 ± 0.01	2.28 ± 0.62	36.4 ± 6.1	1.11 ± 0.35	0.03 ± 0.003
*Trans*-HCA-UA	115 ± 3.6	ND	1.45 ± 0.07	13.3 ± 0.2	251 ± 14.6	17.2 ± 0.5	0.43 ± 0.03

ND = not detected. UA = ursolic acid; OA = oleanolic acid; CA = corosolic acid; MA = maslinic acid; BA = betulinic acid.

**Table 2:** Contents of major triterpenoid acids and esters detected by UPLC-MS in fractions 2C6–2M from the larger scale separation of 5 g POM-ACE (mg/g dry weight ± SD, *n* = 3).

Constituents	2C6	2C9	2D	2F	2G	2H	2J	2K	2M
UA	0.16 ± 0.01	0.59 ± 0.01	52.7 ± 3.5	165 ± 11	556 ± 16	694 ± 9	766 ± 25	661 ± 46	655 ± 28
OA	ND	0.12 ± 0.01	9.66 ± 1.28	214 ± 2	152 ± 5.7	124 ± 13	61 ± 10	42.8 ± 3.1	9.18 ± 0.2
CA	ND	ND	ND	ND	ND	56 ± 1.5	54 ± 1	29.3 ± 0.8	35.1 ± 0.5
MA	ND	ND	ND	ND	ND	30 ± 2.1	12.7 ± 0.3	4.3 ± 0.76	2.43 ± 0.0
BA	ND	ND	0.06 ± 0.01	2.15 ± 0.01	ND	ND	ND	ND	ND
*Cis*-HCA-OA	ND	ND	ND	39.8 ± 1	2.9 ± 0.1	ND	ND	ND	ND
*Cis*-HCA-UA	ND	ND	0.22 ± 0.01	226 ± 16	61.7 ± 2.7	8.6 ± 0.7	2.2 ± 0.2	0.15 ± 0.0	0.17 ± 0.0
*Trans*-HCA-OA	ND	ND	ND	25.5 ± 1	11.3 ± 4.7	ND	ND	ND	ND
*Trans*-HCA-UA	ND	ND	0.20 ± 0.01	182 ± 4	215 ± 2	13 ± 1	2.9 ± 0.2	0.20 ± 0.03	0.17 ± 0.0
MD	ND	ND	ND	ND	ND	ND	ND	0.076 ± 0.01	1.34 ± 0.27
AA	ND	ND	ND	ND	ND	ND	ND	3.81 ± 0.03	4.73 ± 0.012

ND = not detected. UA = ursolic acid; OA = oleanolic acid; CA = corosolic acid; MA = maslinic acid; BA = betulinic acid; MD = madecassic acid; AA = asiatic acid.

**Table 3:** Contents of neutral triterpenoids, phytosterols and tocopherols detected in pomace fractions C, D, 2C1, 2C6, 2C9 and 2D by GC-MS (mg/g dry weight ± SD, *n* = 3).

Constituents	POM-ACE	C	D	2C1	2C6	2C9	2D
Alpha-amyrin	5.7 + 0.3	ND	288 + 29	ND	ND	43 + 2.7	218 + 9.8
Beta-amyrin	3.9 + 0.15	ND	157 + 30	ND	ND	14.5 + 0.12	131 + 7.3
Beta-sitosterol	28 + 2.5	ND	265 + 3	ND	ND	ND	302 + 4.2
Campesterol	10.5 + 0.46	ND	41 + 1.3	ND	ND	ND	10.5 + 0.16
Alpha- tocopherol	1.6 + 0.26	2.09 + 0.3	31 + 5.1	1.5 + 0.4	2.9 + 0.1	1.68 + 0.01	16.5 + 2.7
Beta- tocotrienol	ND	ND	ND	ND	ND	4.1 + 0.96	ND
Taraxasterol	ND	ND	73 + 1.5	ND	ND	6.1 + 0.26	46 + 2.0
Lupeol	ND	ND	ND	ND	ND	2.35 + 0.01	ND

ND = not detected. C and D = fractions from 1 g of POM-ACE; 2C1, 2C6, 2C9, 2D = fractions from 5 g POM-ACE. Other fractions did not show detectable quantities of these analytes.

**Table 4:** Approximate mean percent LOX inhibition (±SD) of activity and calculated IC_50_ for selected POM-ACE fractions and standards.

Standard or Fraction	Inhibition % (125 µg/mL)	IC_50_ (µg/mL)
Corosolic acid	46.1 ± 0.9	271
Ursolic Acid	38.8 ± 1.0	248
2G	72.1 ± 1.4	104
2H	54.0 ± 2.2	136
K	50.4 ± 0.4	200

**Table 5:** Interaction scores (kcal/mol) * for pomace components with target enzymes generated in YASARA/Autodock.

	Caspase-3	Caspase-9	NLRP3
Ursolic acid	7.7	8.6	8.4
Oleanolic acid	9.1	7.9	8.2
Corosolic acid	8.4	8.2	8.5
α-amyrin	7.7	8.7	8.3
β-sitosterol	8.1	7.5	7.3
α-tocopherol	6.6	5.7	5.6

* Positive values indicate favorable interactions in YASARA, opposite of convention.

## Data Availability

The original contributions presented in the study are included in the article and Supplementary Materials; further inquiries can be directed to the corresponding author.

## Supplementary Materials

The following supporting information can be downloaded at: https://www.mdpi.com/article/10.3390/molecules31162853/s1, Figure S1: HPLC-DAD chromatogram of POM-ACE (wavelength = 205 nm) showing profiles of UA, OA, CA, MA, HCA esters of OA and UA; Figure S2: UPLC-MS extracted ion chromatograms showing the major triterpenoid acid and esters in POM-ACE. Top: MA and CA; Middle: OA and UA; Bottom: *trans*- and *cis*-HCA esters of OA and UA respectively. Figure S3: GC-MS total ion chromatogram showing (a) standard mixture of sterols and tocopherols and (b) POM-ACE; Figure S4: UPLC-MS extracted ion chromatogram of beta-sitosterol using method described in Section S2.2; Figure S5: Inhibition of LPS-induced IL-1β secretion in THP-1 cells (model) by treatment with pomace fraction D at concentrations of 1.25–100 µg/mL; Table S1: Major pooled fractions from separation of 1 g POM-ACE; Table S2: Major pooled fractions from separation of 5 g POM-ACE; Table S3: Flow cytometry data for HT-29 cells, untreated control, fractions, and standard; Table S4: IC_50_ data for 24 h treatment of HT-29 cells based on MTT assay; Tables S5–S10: Non-bond interactions of the docked complex of NLRP3 with ursolic acid, corosolic acid, oleanolic acid, amyrin, beta-sitosterol and alpha-tocopherol respectively; Tables S11–S16: Non-bond interactions of the docked complex of caspase-3 with ursolic acid, corosolic acid, oleanolic acid, amyrin, beta-sitosterol and alpha-tocopherol respectively; Tables S17–S22: Non-bond interactions of the docked complex of caspase-9 with ursolic acid, corosolic acid, oleanolic acid, amyrin, beta-sitosterol and alpha-tocopherol respectively. Ref. [73] is cited in Supplementary Materials.

## Abbreviations

The following abbreviations are used in this manuscript:

AA	Asiatic Acid
BA	Betulinic Acid
BS	Beta-Sitosterol
CA	Corosolic Acid
COX	Cyclooxygenase
DAD	Diode-Array Detector
DMSO	Dimethyl Sulfoxide
DW	Dry Weight
EDTA	Ethylenediaminetetraacetic Acid
EI	Electron Ionization
EIC	Extracted Ion Chromatogram
ELISA	Enzyme-Linked Immunosorbent Assay
ESI	Electrospray Ionization
FITC	Fluorescein Isothiocyanate
GC-MS	Gas Chromatography and Mass Spectroscopy
HCA	Hydroxycinnamic Acid
HPLC	High Performance Liquid Chromatography
HT-29	Human Colon Adenocarcinoma Cell Line (Human Tumor-29)
IC_50_	Inhibition Concentration 50%
IL-1β	Interleukin-1 Beta
LOX	Lipoxygenase
LPS	Lipopolysaccharide
*m*/*z*	Mass-to-Charge Ratio
MA	Maslinic Acid
MD	Madecassic Acid
MS	Mass Spectrum
MTT	3-(4,5-Dimethylthiazol-2-yl)-2,5-Diphenyl-Tetrazolium Bromide
NLRP3	NOD-, LRR-, And Pyrin Domain-Containing Protein 3
OA	Oleanolic Acid
PBS	Phosphate-Buffered Saline
PI	Propidium Iodide
PLS	Partial Least Squares
PMA	Phorbol 12-Myristate 13-Acetate
ppm	Parts per Million
RPMI	Roswell Park Memorial Institute
TNF-α	Tumor Necrosis Factor-α
UA	Ursolic Acid
UPLC-MS	Ultra Performance Liquid Chromatography–Mass Spectrometry
YASARA	Yet Another Scientific Artificial Reality Application
