# Supplementary material for: Characterization of Anti-Inflammatory and Anti-Proliferative Triterpenoids and Phytosterols from Cranberry Pomace
Source: Molecules. 2026 Aug 15;31(16):2853. doi: 10.3390/molecules31162853 (PMC13515605; doi:10.3390/molecules31162853)
Supplement: Supplementary file 1 [file molecules-31-02853-s001.zip › molecules-4414150-supplementary.pdf]

Supplementary Materials

Table S1: Major pooled fractions from separation of 1g POM-ACE

Pooled fractions	Assigned name	Quantity (g)	Compounds detected by TLC analysis	Elution solvents and volume
**A1-A15**	A	0.010	Fatty acid, beta carotene	100% n-hexane, 200 mL
**B1-B15**	B	0.010	Fatty acid, beta carotene, lutein ester	100 % n-hexane, 90:10 hex: Et
**C9-C15**	C	0.400	β-carotene, Lutein ester, unknown UV-active	90:10 Hex/EtOAc 200 mL
**D1-F5**	D	0.050	α-amyrin, β-sitosterol, stigasterol, beta carotene	85:15 Hex/Et 250 mL
**F6-H15**	F	0.060	Oleanolic acid, Ursolic acid, UA esters	80:20, 250 mL 70:30 H/Et 250 mL
**I1-J15**	J	0.080	Oleanolic acid, Ursolic acid	60:40 H/Et 250 mL
**K1-M8**	K	0.070	corosolic acid, corosolic acid ester	50:50 H/Et 300 mL
**M9-P7**	M	0.220	Oleanolic acid, corosolic acid, ursolic acid, quercetin	50:45:5 H/Et/M 300 mL

Table S2: Major pooled fractions from separation of 5 g POM-ACE

Pooled fractions	Assigned name	Quantity (g)	Compounds detected by TLC analysis	Solvents and volume
**A1-B30**	2A	0.148	Colorless oil. Likely fatty acids and waxes	100% Hexane, 700 mL, 95:5 (Hex: EtOAc) 900mL
**C1-C5**	2C1	1.449	Yellow oil. Contains beta-carotene, alpha-tocopherol, seed oil components.	95:5 (Hex: EtOAc) 900mL
**C6-C8**	2C6	0.227	Clear oil. Contains seed oil components, beta-sitosterol, alpha-amyrin	95:5 (Hex: EtOAc) 900mL
**C9-C30**	2C9	0.083	Sticky yellow solid. Contains alpha-tocopherol, alpha-amyrin	95:5, 90:10 (Hex: EtOAc), 500 mL each
**D1-E15**	2D	0.274	Sticky yellow solid. Contains beta-sitosterol, alpha-amyrin, tocopherol, ursolic & oleanolic acid	90:10, 85:15 (Hex: EtOAc) 600mL each
**E16-F30**	2F	0.575	Off white solid. Contains MA, CA, OA, UA, HCA esters of OA and UA	80:20, 70:30 (Hex: EtOAc), 600 mL each
**G1-G30**	2G	0.241	Off white solid. Contains MA, CA, OA, UA, HCA esters of OA and UA	60:40 (Hex:EtOAc) 1000mL
**H1-I15**	2H	0.269	Off white solid. Contains UA, OA, MA, CA, unidentified UV active component	60:40, 50:50 (Hex: EtOAc) 1560 mL
**I16-J30**	2J	0.090	Slight greenish solid. Contains UA, OA, CA, MA, UV active component	50:50 (Hex: EtOAc) 1560 mL
**K1-L30**	2K	0.393	Off white solid. Contains UA, OA, MA, CA, unidentified UV active component	50:45:5, 50:30:20 (Hex: EtOAc: MeOH) 1000 mL each
**M1-N25**	2M	0.299	Off white solid. Contains UA, OA, MA, CA, unidentified UV active compound	50:30:20 (Hex: EtOAc: MeOH); 80:20 (EtOAc: MeOH), 1000 mL
**N26-P22**	2P	0.025	Yellowish solid, less triterpenoids & Polyphenol	50:50 (EtOAc:MeOH), 1000 mL; 100% acetone, 300 mL

Section S1 Quantitative analysis of triterpenoids in cranberry pomace extracts by HPLC-DAD.

A reliable characterization method for triterpenoid-rich cranberry pomace extracts by HPLC-DAD was developed based on modification of a published method [1]. POM-ACE extracts were dissolved in methanol, vortexed, ultrasonicated, and filtered (0.45 μm PTFE). Analyses were performed on a Waters Millennium binary HPLC system with two 515 pumps and a 996 PDA detector, using a Phenomenex Synergi Hydro-RP column (250 × 4.6 mm, 4 μm, 80 Å). The mobile phase was 79.8% acetonitrile, 20% water, and 0.2% phosphoric acid, elution technique was isocratic at 0.3 mL/min per pump. Detection was at 205 nm wavelength with a 40 min run time. Injection volume was 20 μL. Triterpenoids were identified in comparison to authentic standards and quantified with a five-point calibration curve. HCA esters were identified based on additional peak UV absorbance at 308-312 nm and comparison to LCMS data [2]. Samples were run in triplicate, results are reported as the mean +/− the standard deviation, and data were processed using Millennium software.

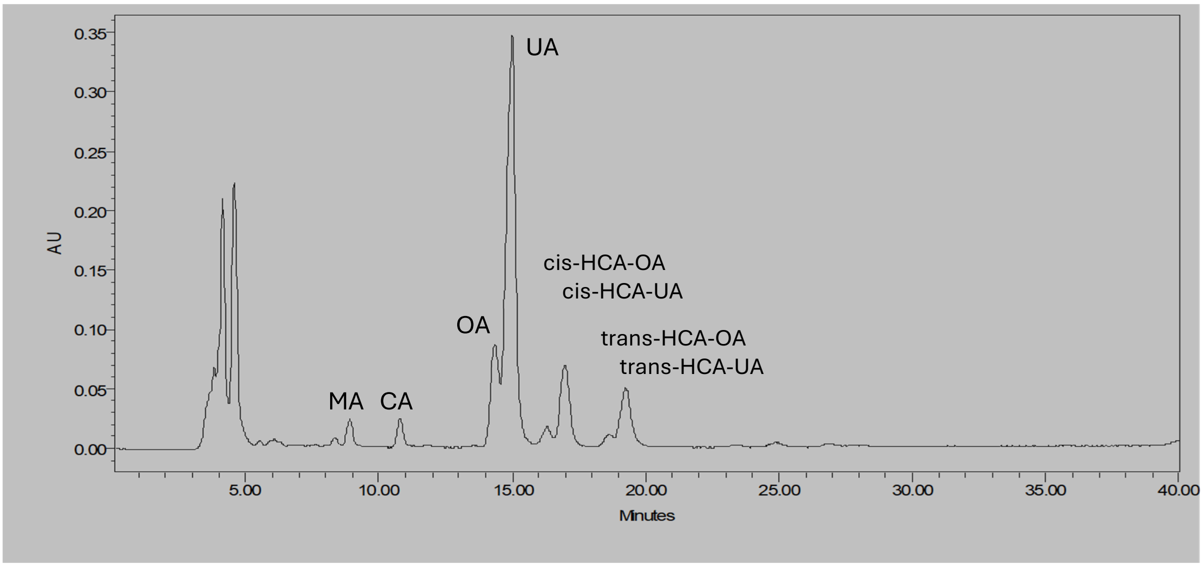

Figure S1: HPLC-DAD chromatogram of POM-ACE (wavelength = 205 nm) showing profiles of UA, OA, CA, MA, *cis*- and *trans*-HCA esters of OA and UA.

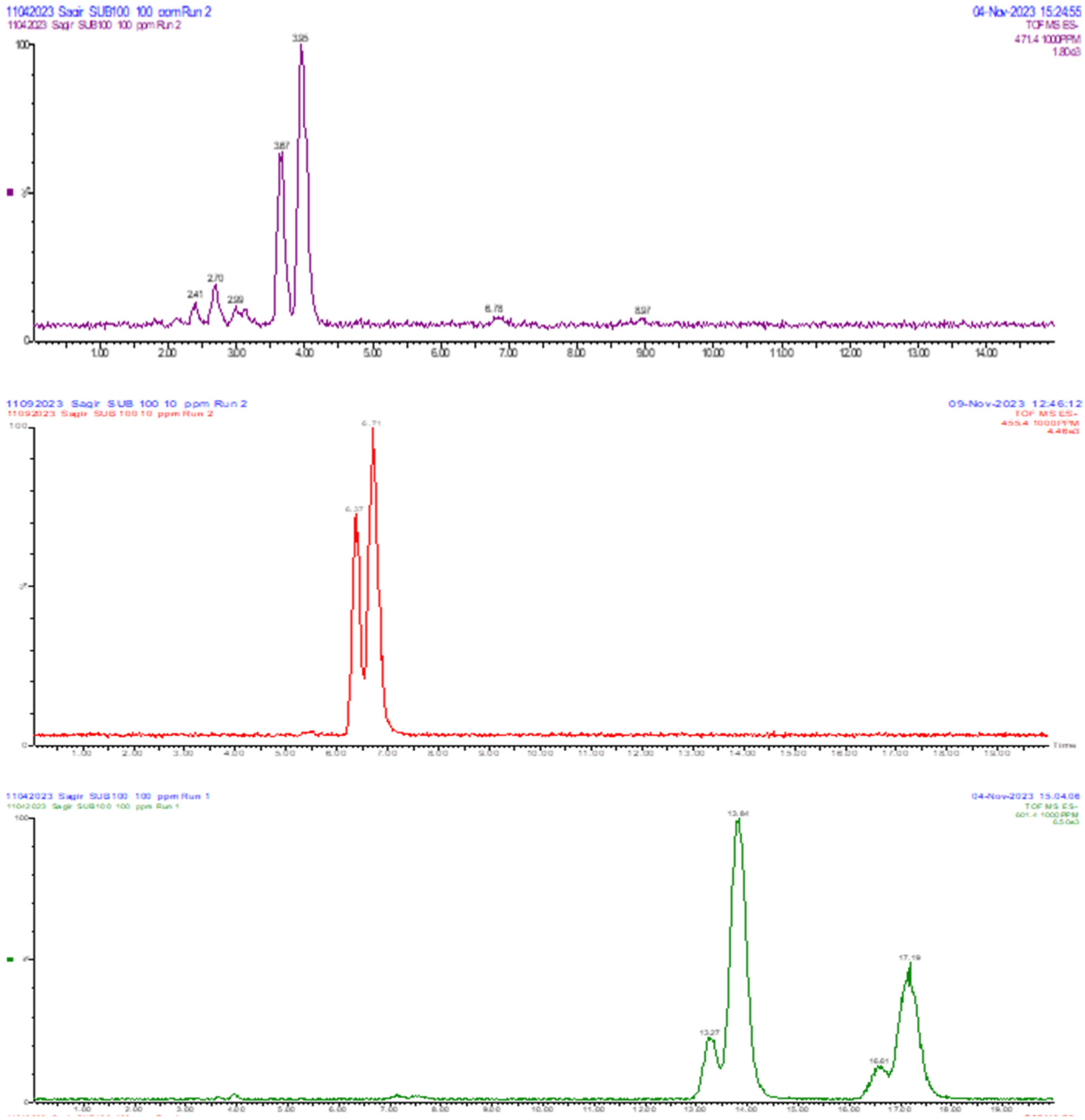

Figure S2: UPLC-MS Extracted Ion chromatograms showing the major triterpenoid acid and esters in POM-ACE. Top: MA and CA; Middle: OA and UA; Bottom: *cis*-HCA esters of OA, cis-HCA esters of UA, trans-HCA esters of OA, and trans-HCA esters of UA.

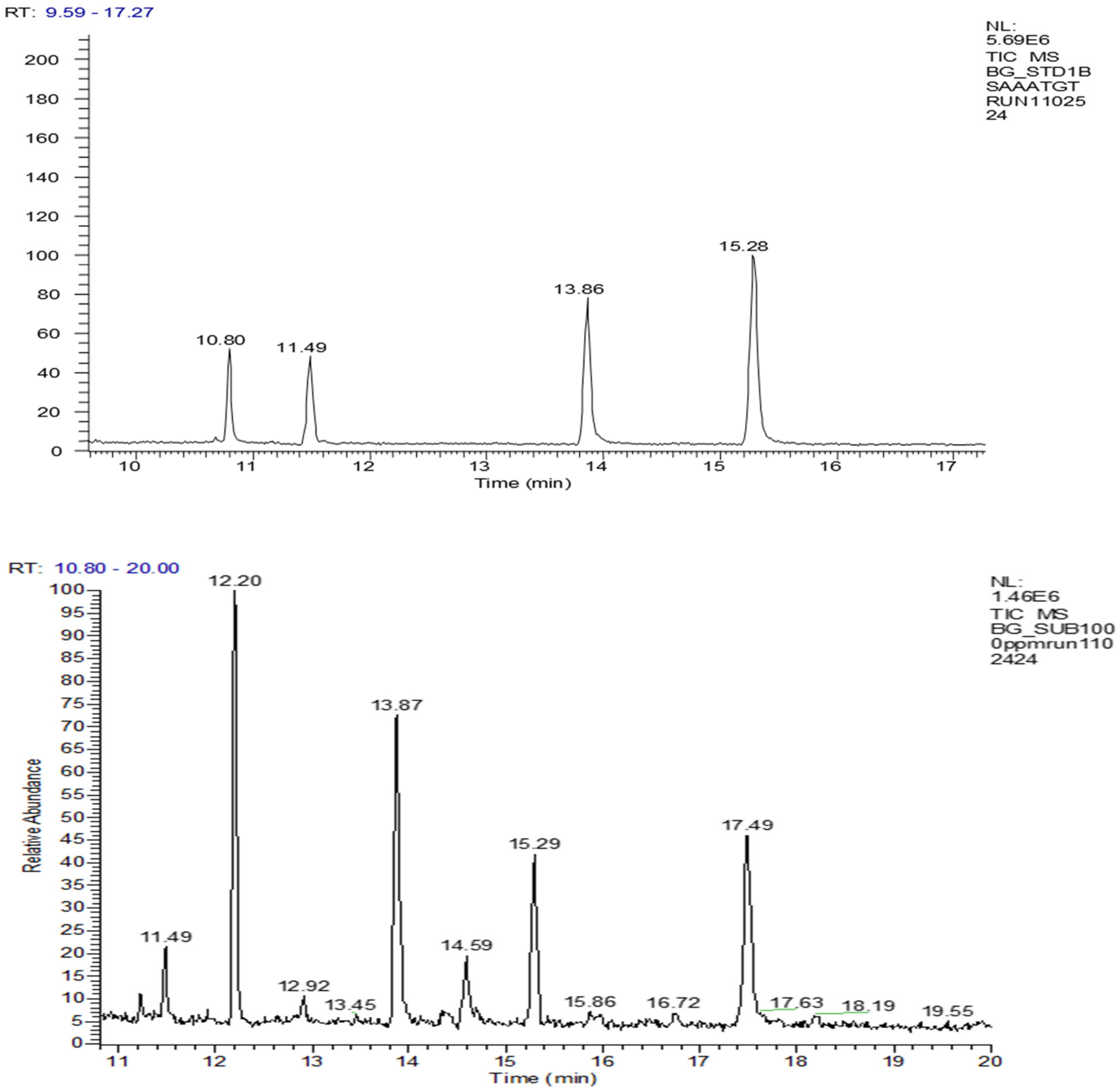

Figure S3: GC-MS total ion chromatogram showing (a) standard mixture of sterols and tocopherols and (b) POM-ACE; peaks are identified at RT = 11.49 min: alpha-tocopherol, RT = 12.20 min: tricontanal, RT = 13.87: beta-sitosterol, RT = 14.59: beta-amyrin, RT =15.29: alpha-amyrin, RT =17.49: tritricontane-12,14-dione.

Section S2: Quantitative analysis of phytosterols in cranberry pomace extracts by UPLC-MS

A UPLC-MS method was developed to determine beta-sitosterol using Waters Xevo QTOF MS and Acquity UPLC instrumentation. POM-ACE and fractions (1–1000 ppm in methanol) were analyzed on a Waters Acquity UPLC BEH C18 column (100×2.1 mm, 1.7 µm) using isocratic elution (30% methanol, 70% acetonitrile; 0.1% formic acid) at 0.3 mL/min for 20 min run time. Injection volume was 1 µL introduced by autosampler, and detection was in positive ESI-MS mode (capillary voltage: 2.5 kV, cone voltage: 160 V, source temperature: 150 °C, desolvation temperature: 350 °C, Cone gas flow (L/h): 50, desolvation gas flow (L/h):600). β-sitosterol was confirmed at m/z 397.5 using an authentic standard. Samples were analyzed in a triplicate manner and quantified using the external standard curve method with commercial standard. Metabolite contents are reported as the mean +/− the standard deviation; data analyses were performed using MassLynx version 4.1.

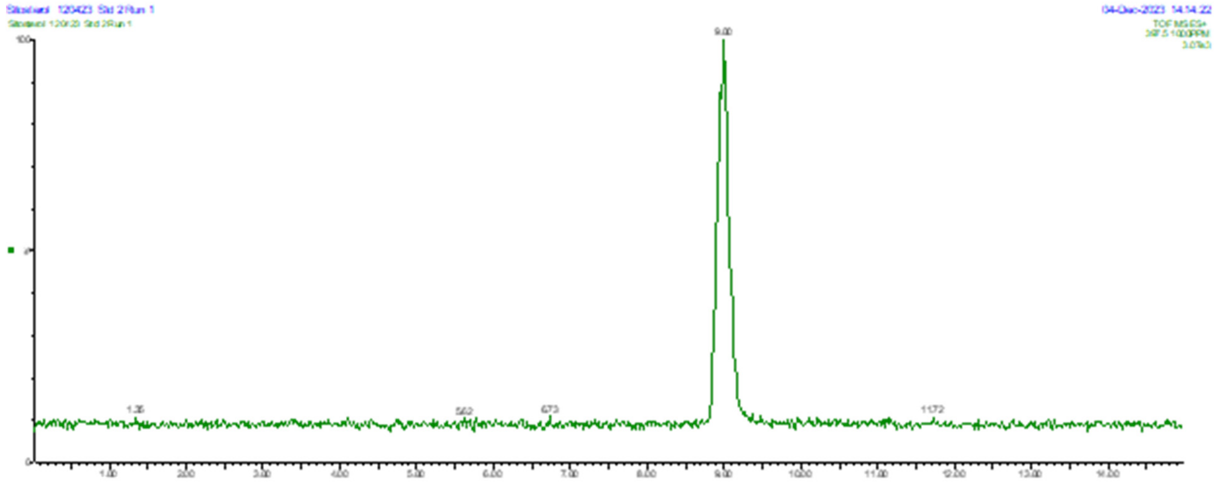

Figure S4: UPLC-MS extracted ion chromatogram of beta sitosterol using method described in

Section S2.2

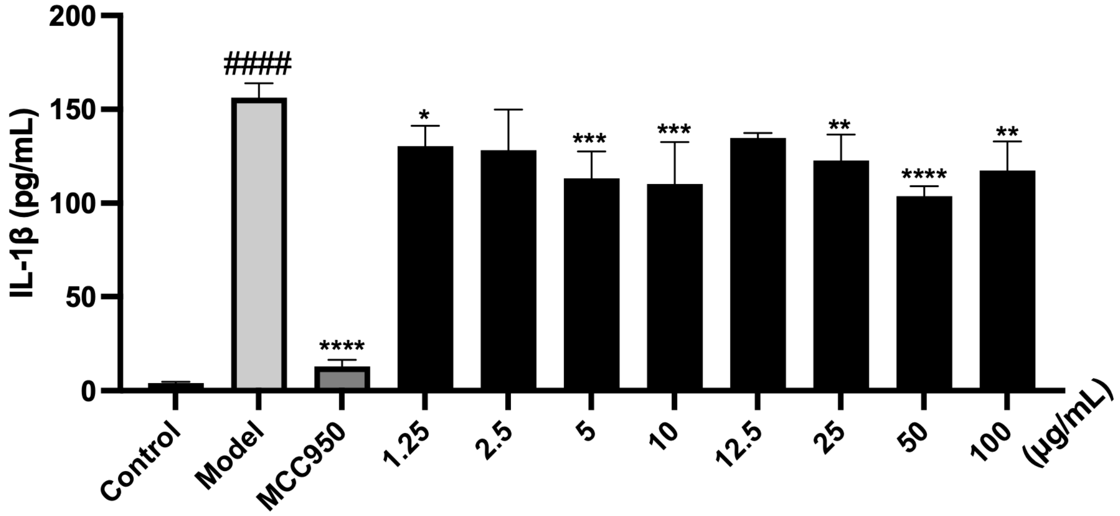

Figure S5: Inhibition of LPS-induced IL-1β secretion in THP-1 cells (model) by treatment with pomace fraction D at concentrations of 1.25 – 100 μg/mL. Significant differences between treatments and model denoted as *p < 0.05, **p < 0.01, ***p < 0.001, ****p < 0.0001. ####p < 0.0001 as compared with the control group.

Table S3: Flow cytometry data for HT-29 cells, untreated control, fractions, and standard

% of cell populations	Untreated HT-29	Ursolic acid-treated	2F treated	2G treated	2K treated
Live cells	96.6	70.4	83.7	87.5	82.8
Early Apoptosis	1.40	14.1	8.55	6.52	10.6
Late apoptosis	1.35	14.3	7.02	5.37	6.03
Necrosis	0.64	0.98	0.64	0.55	0.49

Table S4: IC_{50} data (mean ± standard deviation, n = 3) for 24 h treatment of HT-29 cells with pomace fractions or standards in comparison to untreated control.

Sample	Mean IC_{50} (μg/mL)
Ursolic acid	4.4 ± 2.6
β-Sitosterol	7.1 ± 2.5
POM-ACE	113 ± 0.7
Alpha-tocopherol	291 ± 1.4
F	61 ± 1.6
J	28 ± 2.4
K	26 ± 3.3
M	28 ± 3.2
2C9	200 ± 2.8
2D	63 ± 0.1
2F	12 ± 3
2G	16 ± 1.7
2H	39 ± 0.14
2J	34 ± 3.1
2K	17 ± 0.9
2M	31 ± 2.6

Table S5: Non-bond interactions of the docked complex of NLRP3 with ursolic acid

Compound	Interaction Score (kcal/mol)	Interacting Residue	Distance (Å)	Category	Bond Type
**NLRP3-ursolic**	8.4	SER658	2.58616	Hydrogen Bond	Carbon Hydrogen Bond
		LYS192	4.68588	Hydrophobic	Alkyl
		LEU628	5.39129	Hydrophobic	Alkyl
		LYS192	4.19698	Hydrophobic	Alkyl
		LEU628	4.91488	Hydrophobic	Alkyl
		TYR632	4.76996	Hydrophobic	Pi-Alkyl

YASARA generates a higher positive score to represent a more favorable binding interaction.

Table S6: Non-bond interactions of the docked complex of NLRP3 with corosolic acid

Compound	Interaction Score (kcal/mol)	Interacting Residue	Distance (Å)	Category	Bond Type
**NLRP3-corosolic**	8.5	ARG920	4.6581	Electrostatic	Attractive Charge
		ARG779	2.89586	Hydrogen Bond	Conventional Hydrogen Bond
		ARG779	1.91742	Hydrogen Bond	Conventional Hydrogen Bond
		VAL835	5.35323	Hydrophobic	Alkyl
		VAL892	5.31753	Hydrophobic	Alkyl
		TYR861	5.2336	Hydrophobic	Pi-Alkyl
		TYR861	4.7831	Hydrophobic	Pi-Alkyl
		TYR918	3.66095	Hydrophobic	Pi-Alkyl

YASARA generates a higher positive score to represent a more favorable binding interaction.

Table S7: Non-bond interactions of the docked complex of NLRP3 with oleanolic acid

Compound	Interaction Score (kcal/mol)	Interacting Residue	Distance (Å)	Category	Bond Type
**NLRP3-oleanolic-acid**	8.2	ARG147	4.0283	Electrostatic	Attractive Charge
		TYR143	2.01923	Hydrogen Bond	Conventional Hydrogen Bond
		PRO283	5.37328	Hydrophobic	Alkyl
		LEU57	4.22184	Hydrophobic	Alkyl
		PRO283	5.141	Hydrophobic	Alkyl
		PHE61	4.21045	Hydrophobic	Pi-Alkyl

YASARA generates a higher positive score to represent a more favorable binding interaction.

Table S8: Non-bond interactions of the docked complex of NLRP3 with amyrin

Compound	Interaction Score (kcal/mol)	Interacting Residue	Distance (Å)	Category	Bond Type
**NLRP3-amyrin**	8.3	HIS685	1.8412	Hydrogen Bond	Conventional Hydrogen Bond
		PHE406	4.94849	Hydrophobic	Pi-Alkyl
		PHE406	3.48209	Hydrophobic	Pi-Alkyl

YASARA generates a higher positive score to represent a more favorable binding interaction.

Table S9: Non-bond interactions of the docked complex of NLRP3 with beta-sitosterol

Compound	Interaction Score (kcal/mol)	Interacting Residue	Distance (Å)	Category	Bond Type
**NLRP3-sitosterol**	7.3	TRP478	2.7124	Hydrophobic	Pi-Sigma
		PRO554	5.34184	Hydrophobic	Alkyl
		PRO554	3.75663	Hydrophobic	Alkyl
		VAL560	4.27421	Hydrophobic	Alkyl
		TRP478	4.14683	Hydrophobic	Pi-Alkyl
		TRP478	5.03151	Hydrophobic	Pi-Alkyl
		PHE568	4.89258	Hydrophobic	Pi-Alkyl

YASARA generates a higher positive score to represent a more favorable binding interaction.

Table S10: Non-bond interactions of the docked complex of NLRP3 with alpha-tocopherol

Compound	Interaction Score (kcal/mol)	Interacting Residue	Distance (Å)	Category	Bond Type
**NLRP3-tocopherol**	5.6	LEU551	4.87795	Hydrophobic	Alkyl
		LYS552	4.93206	Hydrophobic	Alkyl
		VAL560	4.65964	Hydrophobic	Alkyl
		LYS552	4.07828	Hydrophobic	Alkyl
		TRP478	4.35116	Hydrophobic	Pi-Alkyl
		TRP478	5.19878	Hydrophobic	Pi-Alkyl
		TRP478	5.17157	Hydrophobic	Pi-Alkyl
		TRP478	4.1973	Hydrophobic	Pi-Alkyl
		TRP478	4.61596	Hydrophobic	Pi-Alkyl
		HIS492	4.7531	Hydrophobic	Pi-Alkyl

YASARA generates a higher positive score to represent a more favorable binding interaction.

Table S11: Non-bond interactions of the docked complex of caspase-3 with ursolic acid

Compound	Interaction Score (kcal/mol)	Interacting Residue	Distance (Å)	Category	Bond Type
**caspase-3-ursolic**	8.6	ASN208	2.37585	Hydrogen Bond	Conventional Hydrogen Bond

YASARA generates a higher positive score to represent a more favorable binding interaction.

Table S12: Non-bond interactions of the docked complex of caspase-3 with oleanolic acid

Compound	Interaction Score (kcal/mol)	Interacting Residue	Distance (Å)	Category	Bond Type
**caspase-3-oleanolic-acid**	9.1	ARG207	2.55823	Hydrogen Bond	Conventional Hydrogen Bond
		SER209	2.6186	Hydrogen Bond	Conventional Hydrogen Bond
		TYR204	5.23692	Hydrophobic	Pi-Alkyl
		TRP206	4.50807	Hydrophobic	Pi-Alkyl
		PHE256	4.34434	Hydrophobic	Pi-Alkyl

YASARA generates a higher positive score to represent a more favorable binding interaction.

Table S13: Non-bond interactions of the docked complex of caspase-3 with corosolic acid

Compound	Interaction Score (kcal/mol)	Interacting Residue	Distance (Å)	Category	Bond Type
**Caspase-3-corosolic acid**	8.4	ARG207	4.96497	Electrostatic	Attractive Charge
		ASN208	2.30247	Hydrogen Bond	Conventional Hydrogen Bond
		PHE250	4.92191	Hydrophobic	Pi-Alkyl
		PHE256	4.91864	Hydrophobic	Pi-Alkyl
		PHE256	4.11372	Hydrophobic	Pi-Alkyl

YASARA generates a higher positive score to represent a more favorable binding interaction.

Table S14: Non-bond interactions of the docked complex of caspase-3 with amyrin

Compound	Interaction Score (kcal/mol)	Interacting Residue	Distance (Å)	Category	Bond Type
**caspase-3-amyrin**	8.7	ARG207	2.18839	Hydrogen Bond	Conventional Hydrogen Bond
		PHE250	5.20741	Hydrophobic	Pi-Alkyl
		TRP206	4.94278	Hydrophobic	Pi-Alkyl

YASARA generates a higher positive score to represent a more favorable binding interaction.

Table S15: Non-bond interaction of the docked complex of caspase-3 with sitosterol

Compound	Interaction Score (kcal/mol)	Interacting Residue	Distance (Å)	Category	Bond Type
**caspase-3-sitosterol**	8.1	TYR204	5.29395	Hydrophobic	Pi-Alkyl
		TRP206	4.70073	Hydrophobic	Pi-Alkyl
		PHE256	4.49757	Hydrophobic	Pi-Alkyl
		PHE256	4.27696	Hydrophobic	Pi-Alkyl

YASARA generates a higher positive score to represent a more favorable binding interaction.

Table S16: Non-bond interactions of the docked complex of caspase-3 with alpha tocopherol

Compound	Interaction Score (kcal/mol)	Interacting Residue	Distance (Å)	Category	Bond Type
**caspase-3-tocopherol**	6.6	ARG207	4.91688	Hydrophobic	Alkyl
		TRP206	4.78722	Hydrophobic	Pi-Alkyl
		TRP206	4.98119	Hydrophobic	Pi-Alkyl
		TRP206	5.04071	Hydrophobic	Pi-Alkyl
		TRP214	4.63118	Hydrophobic	Pi-Alkyl
		TRP214	5.10767	Hydrophobic	Pi-Alkyl
		PHE250	5.31389	Hydrophobic	Pi-Alkyl
		PHE256	4.52231	Hydrophobic	Pi-Alkyl
		PHE256	5.34942	Hydrophobic	Pi-Alkyl
		PHE256	5.44959	Hydrophobic	Pi-Alkyl
		PHE256	4.96946	Hydrophobic	Pi-Alkyl

YASARA generates a higher positive score to represent a more favorable binding interaction.

Table S17: Non-bond interactions of the docked complex of caspase-9 with ursolic acid

Compound	Interaction Score (kcal/mol)	Interacting Residue	Distance (Å)	Category	Bond Type
**caspase-9-ursolic**	7.8	ARG146	5.06473	Electrostatic	Attractive Charge
		ARG146	2.69605	Hydrogen Bond	Conventional Hydrogen Bond
		ARG146	2.86101	Hydrogen Bond	Conventional Hydrogen Bond
		LYS401	2.78157	Hydrogen Bond	Carbon Hydrogen Bond
		ARG146	5.07178	Hydrophobic	Alkyl
		ILE154	4.67966	Hydrophobic	Alkyl

YASARA generates a higher positive score to represent a more favorable binding interaction.

Table S18: Non-bond interactions of the docked complex of caspase-9 with corosolic acid

Compound	Interaction Score (kcal/mol)	Interacting Residue	Distance (Å)	Category	Bond Type
**caspase-9-corosolic**	8.2	ASN263	3.01083	Hydrogen Bond	Conventional Hydrogen Bond
		GLY275	2.90635	Hydrogen Bond	Carbon Hydrogen Bond
		VAL262	3.99201	Hydrophobic	Alkyl
		VAL262	4.60769	Hydrophobic	Alkyl
		ILE327	5.35467	Hydrophobic	Alkyl
		PHE240F	4.69289	Hydrophobic	Pi-Alkyl

YASARA generates a higher positive score to represent a more favorable binding interaction.

Table S19: Non-bond interactions of the docked complex of caspase-9 with oleanolic acid

Compound	Interaction Score (kcal/mol)	Interacting Residue	Distance (Å)	Category	Bond Type
**caspase-9-oleanolic-acid**	7.9	LYS290	4.35091	Electrostatic	Attractive Charge
		ILE382	2.39807	Hydrogen Bond	Conventional Hydrogen Bond
		TYR383	1.98403	Hydrogen Bond	Conventional Hydrogen Bond
		TRP340	5.40182	Hydrophobic	Pi-Alkyl

YASARA generates a higher positive score to represent a more favorable binding interaction.

Table S20: Non-bond interactions of the docked complex of caspase-9 with alpha amyrin

Compound	Interaction Score (kcal/mol)	Interacting Residue	Distance (Å)	Category	Bond Type
**caspase-9-amyrin**	8.7	ILE382	4.64941	Hydrophobic	Alkyl
		TRP340	4.39888	Hydrophobic	Pi-Alkyl
		TRP340	4.96887	Hydrophobic	Pi-Alkyl
		TRP340	3.89869	Hydrophobic	Pi-Alkyl
		TRP348	4.89273	Hydrophobic	Pi-Alkyl

YASARA generates a higher positive score to represent a more favorable binding interaction.

Table S21: Non-bond interactions of the docked complex of caspase-9 with sitosterol

Compound	Interaction Score (kcal/mol)	Interacting Residue	Distance (Å)	Category	Bond Type
**caspase-9-sitosterol**	7.5	THR323	2.54993	Hydrogen Bond	Conventional Hydrogen Bond
		THR323	2.93538	Hydrogen Bond	Carbon Hydrogen Bond
		PRO324	2.3918	Hydrogen Bond	Carbon Hydrogen Bond
		PRO240	5.32525	Hydrophobic	Alkyl
		PRO240	5.34825	Hydrophobic	Alkyl
		PRO322	4.14534	Hydrophobic	Alkyl
		PRO240	4.76517	Hydrophobic	Alkyl
		PRO335	4.63757	Hydrophobic	Alkyl
		HIS240	5.22516	Hydrophobic	Pi-Alkyl
		HIS240	4.70862	Hydrophobic	Pi-Alkyl
		PHE334	4.9505	Hydrophobic	Pi-Alkyl

YASARA generates a higher positive score to represent a more favorable binding interaction.

Table S22: Non-bond interactions of the docked complex of caspase-9 with tocopherol

Compound	Interaction Score (kcal/mol)	Interacting Residue	Distance (Å)	Category	Bond Type
**caspase-9-tocopherol**	5.7	VAL262	5.29675	Hydrophobic	Alkyl
		VAL262	4.93674	Hydrophobic	Alkyl
		ILE327	5.28678	Hydrophobic	Alkyl
		VAL262	4.37248	Hydrophobic	Alkyl
		ILE327	4.61737	Hydrophobic	Alkyl
		PHE240F	5.49423	Hydrophobic	Pi-Alkyl
		PHE240F	4.92491	Hydrophobic	Pi-Alkyl

YASARA generates a higher positive score to represent a more favorable binding interaction.

References:

1. Vilkickyte, G.; Raudone, L. Optimization, Validation and Application of HPLC-PDA Methods for Quantification of Triterpenoids in Vaccinium Vitis-Idaea L. *Molecules* **2021**, *26*, doi:10.3390/molecules26061645.

2. Xue, L.; Carreiro, B.; Mia, M.S.S.; Paetau-Robinson, I.; Khoo, C.; Neto, C. Pentacyclic Triterpenoid Content in Cranberry Raw Materials and Products. *Foods* **2024**, *13*, 3136, doi:10.3390/foods13193136.
